# Treatment with inhaled Argon: a systematic review of pre-clinical and clinical studies with meta-analysis on neuroprotective effect

**DOI:** 10.1016/j.ebiom.2024.105143

**Published:** 2024-04-30

**Authors:** Giulia Merigo, Gaetano Florio, Fabiana Madotto, Aurora Magliocca, Ivan Silvestri, Francesca Fumagalli, Marianna Cerrato, Francesca Motta, Daria De Giorgio, Mauro Panigada, Alberto Zanella, Giacomo Grasselli, Giuseppe Ristagno

**Affiliations:** a Department of Biomedical Sciences for Health, University of Milan, Milan, Italy; b Department of Anesthesiology, Intensive Care and Emergency, Fondazione IRCCS Ca’ Granda Ospedale Maggiore Policlinico, Milan, Italy; c Department of Pathophysiology and Transplantation, University of Milan, Milan, Italy; d Department of Acute Brain and Cardiovascular Injury, Istituto di Ricerche Farmacologiche Mario Negri IRCCS, Milan, Italy

**Keywords:** Argon, Noble gas, Neuroprotection, Organ protection, Meta-analysis

## Abstract

**Background:**

Argon (Ar) has been proposed as a potential therapeutic agent in multiple clinical conditions, specifically in organ protection. However, conflicting data on pre-clinical models, together with a great variability in Ar administration protocols and outcome assessments, have been reported. The aim of this study was to review evidence on treatment with Ar, with an extensive investigation on its neuroprotective effect, and to summarise all tested administration protocols.

**Methods:**

Using the PubMed database, all existing pre-clinical and clinical studies on the treatment with Ar were systematically reviewed (registration: https://doi.org/10.17605/OSF.IO/7983D). Study titles and abstracts were screened, extracting data from relevant studies post full-text review. Exclusion criteria included absence of full text and non-English language. Furthermore, meta-analysis was also performed to assess Ar potential as neuroprotectant agent in different clinical conditions: cardiac arrest, traumatic brain injury, ischemic stroke, perinatal hypoxic-ischemic encephalopathy, subarachnoid haemorrhage. Standardised mean differences for neurological, cognitive and locomotor, histological, and physiological measures were evaluated, through appropriate tests, clinical, and laboratory variables. *In vivo* studies were evaluated for risk of bias using the Systematic Review Center for Laboratory Animal Experimentation tool, while *in vitro* studies underwent assessment with a tool developed by the Office of Health Assessment and Translation.

**Findings:**

The systematic review detected 60 experimental studies (16 *in vitro*, 7 *ex vivo*, 31 *in vivo*, 6 with both *in vitro* and *in vivo*) investigating the role of Ar. Only one clinical study was found. Data from six *in vitro* and nineteen *in vivo* studies were included in the meta-analyses. In pre-clinical models, Ar administration resulted in improved neurological, cognitive and locomotor, and histological outcomes without any change in physiological parameters (i.e., absence of adverse events).

**Interpretation:**

This systematic review and meta-analysis based on experimental studies supports the neuroprotective effect of Ar, thus providing a rationale for potential translation of Ar treatment in humans. Despite adherence to established guidelines and methodologies, limitations in data availability prevented further analyses to investigate potential sources of heterogeneity due to study design.

**Funding:**

This study was funded in part by Italian Ministry of Health-Current researchIRCCS and by Ministero della Salute Italiano, Ricerca Finalizzata, project no. RF 2019-12371416.

**Research in context**

**Evidence before this study**

An increasing number of experimental studies have showed the beneficial effects of the noble gas Argon (Ar), mainly as neuroprotectant agent after an ischemic event. However, wide variability exists in the Ar administration protocols used and in outcomes assessed among different experimental models. Recognising the need to establish a comprehensive overview of the organ protective properties of Ar, a systematic PubMed search was conducted to consolidate and enhance the researchers’ understanding of the subject. As a result, 61 studies and several narrative reviews were identified, laying a solid foundation for this study. Two systematic reviews with meta-analysis are also available but limited to a few experimental models and a comparison of noble gases (i.e., xenon, helium).

**Added value of this study**

The present study provides a comprehensive systematic review of all the existing pre-clinical and clinical evidence on Ar treatment and of all administration/treatment protocols. Furthermore, a meta-analysis assesses Ar neuroprotective effect both *in vitro* and *in vivo* settings, also considering pre-clinical models of different clinical conditions, such as cardiac arrest (CA), traumatic brain injury, ischemic stroke, perinatal hypoxic-ischemic encephalopathy, subarachnoid haemorrhage. These data support the use of Ar as neuroprotectant agent and confirm the safety of the treatment, showing no detrimental effects on haemodynamics, metabolism, and gas exchange.

**Implications of all the available evidence**

The evidence on Ar neuroprotective effect outlined by this systematic review with meta-analysis supports its clinical transition. Because of the low heterogeneity in the administration protocol of Ar across pre-clinical studies, adequate evidence suggests that CA is an optimal candidate for a clinical trial. Indeed, the CardioPulmonary Resuscitation with Argon trial is a just started multicentre, phase I/II, randomised, controlled, single blinded study evaluating safety and feasibility of Ar in patients resuscitated from out-of-hospital CA (NCT05482945). Nevertheless, the experimental evidence provided by this review extends beyond CA to explore the neuroprotective effects of Ar in other clinical conditions.

## Introduction

In recent years, the noble gas Argon (Ar) is gaining growing interest as a potential therapeutic agent for several clinical conditions, due to its multiple properties (i.e., upregulation of pro-survival genes and downregulation of pro-apoptotic ones). Discovered in the late 19th century and named “Argon” from the Greek word “ᾱ̓ργός”, meaning “inactive” or “lazy” in reference to its low reactivity, Ar is the most abundant noble gas, constituting nearly 0.93% of the air composition. It is colourless, tasteless, odourless, non-corrosive, non-flammable, and nontoxic.

Despite being traditionally considered chemically inert owing to its complete electron valence shell, recent evidence suggests significant biological effects of Ar. Early studies noted narcotic effects associated with Ar in divers, corroborated by subsequent investigations. Beyond its narcotic properties, evidence of organ protection has emerged since the early 2000s, concomitant with the investigation of its underlying molecular mechanisms.

Multiple *in vivo* studies have demonstrated Ar efficacy as an organ protectant in various animal models, although some studies have reported neutral or negative results. Antonova et al. have recently reported the existence of conflicting data regarding Ar treatment protocols employed in pre-clinical studies, emphasising the need for a systematic review comparing the different treatment regimens.^1^

Several reviews have summarised noble gases neuroprotective effects in animal models, with two of them specifically focusing on Ar properties.^2^, ^3^, ^4^, ^5^, ^6^, ^7^ A systematic review with meta-analysis published in 2016 investigated the effect of noble gases in ischemia and reperfusion injury (IRI) in pre-clinical models and included seven studies testing Ar treatment.^8^ A more recent systematic review with meta-analysis supported the neuroprotective effect of Ar and Xenon on acquired brain injuries in pre-clinical models of three clinical conditions, i.e., cardiac arrest (CA), ischemic stroke and traumatic brain injury (TBI).^9^ However, a comprehensive assessment of Ar’s role as neuroprotectant in multiple clinical conditions is still lacking.

The present manuscript aims to systematically review existing pre-clinical and clinical evidence on Ar, focusing on its potential therapeutic applications in diverse clinical conditions. Acknowledging the heterogeneity in study design of available literature, this systematic review summarises all tested Ar treatment protocols to aid researchers in selecting appropriate experimental models and determining promising directions for future studies on specific clinical conditions.^1^

Furthermore, with a specific focus on Ar’s neuroprotective effects, a meta-analysis, both on *in vivo* and *in vitro* findings, was conducted to assess Ar efficacy in pre-clinical models of distinct clinical conditions: CA, TBI, ischemic stroke, perinatal hypoxic-ischemic encephalopathy (perinatal HIE), and subarachnoid haemorrhage (SAH).

## Methods

### Search strategy and study selection

The systematic review with meta-analysis was conducted in accordance with the Preferred Reporting Items for Systematic Reviews and Meta-analysis (PRISMA),^10^ as reported in Supplemental Tables S1 and S2. The study protocol was registered with the Open Science Foundation Registries (Registration DOI: https://doi.org/10.17605/OSF.IO/7983D).

On March 15th, 2024, literature was searched in the PubMed database using two distinct algorithms (Supplemental material). The first algorithm (Supplemental Table S3) was applied to identify clinical and pre-clinical, *in vivo,* and *ex-vivo,* studies investigating the potential protective effects of Ar under abovementioned clinical conditions. The second algorithm (Supplemental Table S4) was used to detect relevant *in vitro* studies examining the potential effects of Ar on cellular mechanisms. The two algorithms were combined to comprehensively identify all pertinent scientific literature of interest (Supplemental Table S5). A snowball search was also performed to identify additional studies by searching the reference lists of publications eligible for full-text review. Duplicate entries from the retrieved literature were removed.

Two researchers (GM with expertise in pharmaceutical biotechnology and GF in medical science) autonomously assessed titles and abstracts, reaching consensus through discussion on articles to be screened for full-text examination and data collection. All articles reporting relevant data on the topic and detailing experimental studies were considered. The decision to include or exclude *in-vivo*, *ex-vivo*, and *in-vitro* studies was independent of the sex of the humans, animals, cells, or tissues involved. The exclusion criteria were the absence of the full text and non-English language. Further conflicts were resolved with the corresponding author’s (GR) involvement.

### Data collection

From all eligible articles, the following information were recorded: PMID number, DOI reference, name of the first author, publication year, title, aim, primary and secondary endpoints, model, description of Ar treatment and control treatment (i.e., composition, percentages, and duration), conditioning (pre or post), histopathology, functionality, biochemistry, involved cell/tissue, summary of results and conclusion. The information for both *ex vivo* and *in vivo* studies, were supplemented by providing additional details. This included information on animal models (such as species, age, sex, and weight), study design, clinical conditions and induction method, total number of animals, number of compared groups, number of animals in each group (experimental and control). *In vitro* and *in vivo* studies, neuroprotective outcomes included descriptions, observation times, directions, and statistical measures. These measures encompassed relative and absolute frequency, mean, standard deviation, median, first quartile, third quartile, minimum and maximum observed values, along with any missing observations. For each eligible study intended for use in the meta-analysis, in cases where numerical values were represented graphically, data were extracted from calibrated digitalised plots using a web-based plot digitiser tool (WebPlotDigitizer version 4.6).^11^

Microsoft Excel (Microsoft, Office 365, Redmond, WA, USA) was used for data collection, distinguishing *in vitro* studies from other types of studies. The quality evaluation process to ensure the reliability and accuracy of collected data was conducted collaboratively by the two researchers responsible for screening the literature (GM and GF) and the statistician (FM).

### Quality assessment

Articles eligible for inclusion in the meta-analyses (*in vivo* and *in vitro* studies) for the neuroprotective effects of Ar were independently assessed for quality by two reviewers (GM and GF) and differences in scoring were resolved through discussion with the corresponding author (GR). In detail, Systematic Review Center for Laboratory animal Experimentation (SYRCLE) tool for animal study was used to assess the risk of bias for *in vivo* studies.^12^ Ten items were assessed (yes, no, unclear) to evaluate selection bias, performance bias, attrition bias, detection bias, reporting bias and other possible biases. In assessing *in vitro* studies, the absence of validated tools in this setting led to the use of the risk of bias tool developed by the Office of Health Assessment and Translation (OHAT). This tool employs a comprehensive classification system, comprising five distinct responses (definitely low, probably low, probably high, definitely high, or not reported) for each risk of bias domain.

### Statistics

The neuroprotective effect of Ar was assessed through the analysis of cell protection indexes (cell viability and trauma intensity) *in vitro* studies, and *in vivo* studies multiple measures were examined to provide a comprehensive description across various domains. These domains encompass functional aspects (including neurological, cognitive and locomotor measures) physiological parameters such as haemodynamic, metabolic, and respiratory exchange metrics, as well as histological markers, specifically focusing on neurodegeneration and neuroinflammation (Supplemental Table S6).

To estimate the effect size of Ar neuroprotection, meta-analysis was conducted excluding experimental groups where Ar was administered in combination with other therapies (i.e., hypothermia) and for each endpoint, the standardised mean difference (SMD) with small-sample correction (Hedges’ g) and 95% confidence interval (CI) were estimated. All effect sizes were consistently coded in the same direction (higher effect sizes mean better outcomes in the treatment group). Moreover, the method for unknown non-normal distributions approach was applied to estimate the sample mean and standard deviation from a study that presented median, sample size, quartiles and/or minimum and maximum values.^13^ If a control group served multiple experimental groups, the total number of control animals was divided by the number of experimental groups.^14^ If an article did not allow for the estimation of at least one effect size, it was excluded from the analysis. For each outcome, a random-effects model was adopted, using the inverse variance methods for weights, the restricted maximum likelihood estimator for the variance (tau). Heterogeneity across studies was estimated using Higgins inconsistency index (I^2^).

When examining a specific outcome, if a study contributed more than one effect size (different endpoints to measure the same outcome), a three-level nested random effects model was applied. All measures (level 1) related to the same outcome (level 2) reported in each study (level 3) were included in the nested model structure, in order to account for the inherent dependence between units of analysis and to quantify the variability associated with differences within studies and between studies.^15^

Forest plots were used to display the results graphically, with square area indicating study weight.

To assess the robustness of the meta-analysis findings and investigate heterogeneity, a sensitivity analysis was conducted, excluding studies with low scores on the quality assessment scale. Moreover, for those outcomes where multiple repeated measures were present (cognitive and locomotor function, neurodegeneration, and inflammation outcomes), the pooled effects were also estimated by removing extreme effect sizes (outliers).^16^ Effect size were defined as outliers when their 95% CI lies outside the 95% CI of the pooled effect.

In order to assess potential variations in the neuroprotective effects of Ar depending on its concentration within the mixture *in vivo* studies, a subgroup meta-analysis was conducted classifying studies based on the percentage of Ar concentration (Ar < 70% and Ar ≥ 70%). In the *in vitro* meta-analysis, a similar approach was employed, using the techniques applied for inducing cell injury (oxygen glucose deprivation, traumatic brain injury) to discern potential variations in the Ar neuroprotective effects.

Due to the limited number of studies included in each statistical model, additional potential sources of heterogeneity were not explored (such as the study quality level, characteristics of Ar treatment protocol). For the same reason, the risk of bias associated with missing results of each outcome (reporting bias) was visually assessed using funnel plots, while statistical testing for funnel plot asymmetry was only feasible for outcomes with a minimum of ten effects.

All statistical tests were two-sided, and analysis was mainly conducted with “meta” and “metafor” packages of R version 4.3.2 (The R Project for Statistical Computing; http://www.r-project.org/).

### Ethics

Institutional ethical approval was not required for this systematic review and meta-analysis.

### Role of funders

This study was funded in part by Italian Ministry of Health–Current research IRCCS and by Ministero della Salute Italiano, Ricerca Finalizzata, project no. RF 2019-12371416. The funding entities played no role in the study’s design, data collection and interpretation, report writing, or the decision to submit the manuscript for publication.

## Results

**Fig. 1:**
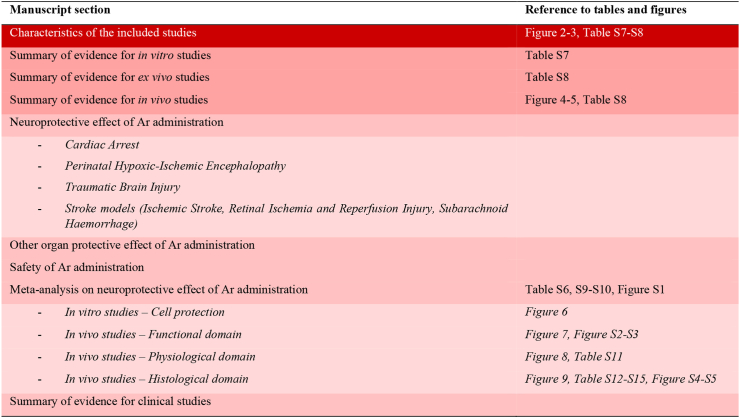
**Framework of findings**.

Due to its complexity, a schematic view of our findings is reported on Fig. 1.

### Characteristics of the included studies

**Fig. 2:**
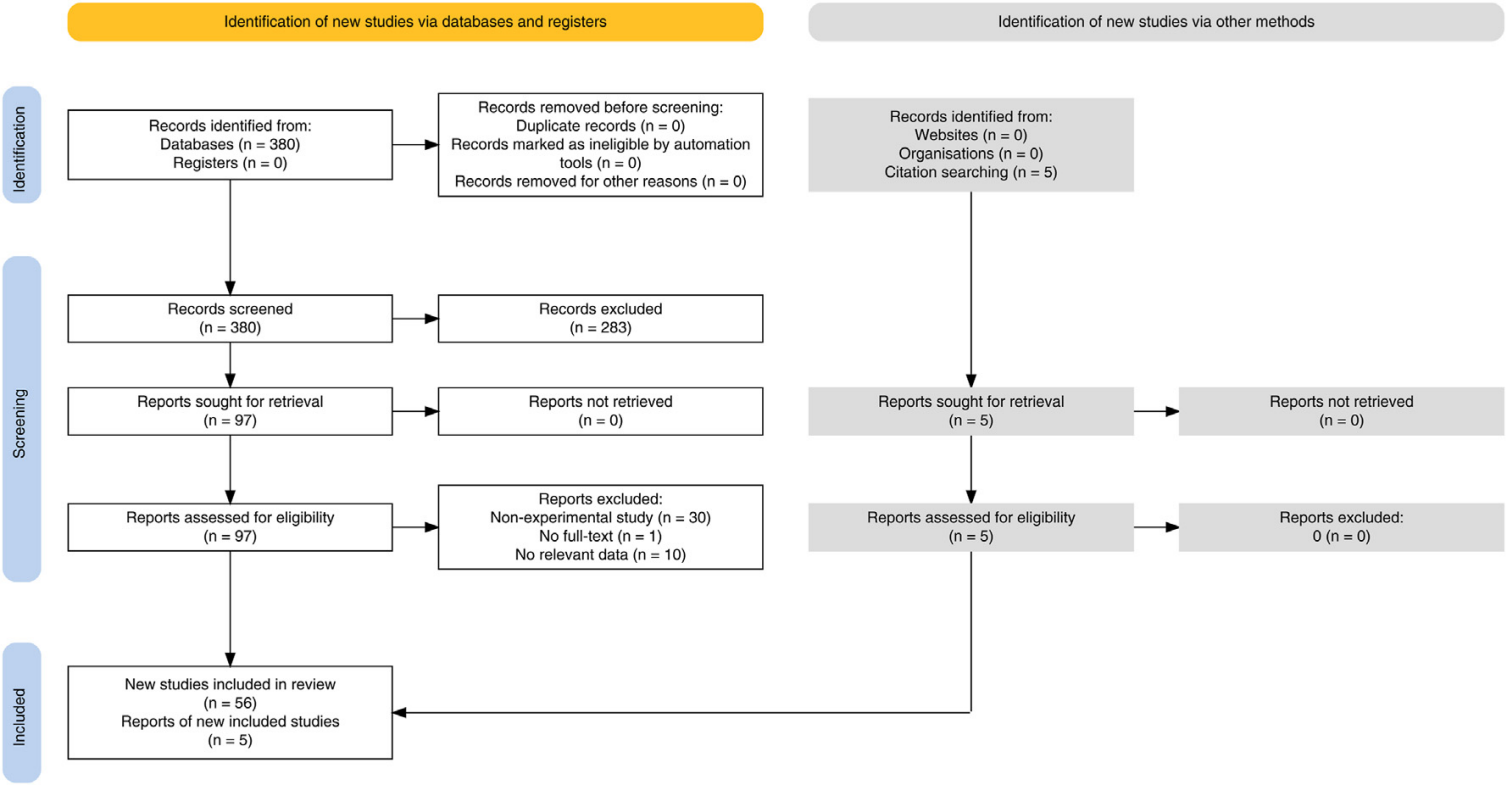
**PRISMA flow diagram for literature search and study selection process of the systematic review**.

The selection process identified 380 studies (Fig. 2). After removing duplicates and conducting title and abstract screening, 97 studies underwent a thorough full-text assessment. Ultimately, 56 studies, published between 2005 and 2024, met inclusion criteria for the systematic review. Additionally, five publications were included, based on references inspection.

Of the 61 selected studies, only one was a clinical study,^17^ while the remaining were pre-clinical studies (Supplemental Tables S7 and S8). There were 16 *in vitro* studies, seven *ex vivo* studies, and 37 *in vivo* studies (six of which also included *in vitro* experiments).^1^^,^^18^, ^19^, ^20^, ^21^, ^22^, ^23^, ^24^, ^25^, ^26^, ^27^, ^28^, ^29^, ^30^^,^^31^, ^32^, ^33^, ^34^, ^35^, ^36^, ^37^, ^38^, ^39^, ^40^, ^41^, ^42^, ^43^, ^44^, ^45^, ^46^, ^47^, ^48^, ^49^, ^50^^,^^51^, ^52^, ^53^, ^54^, ^55^, ^56^, ^57^, ^58^, ^59^, ^60^, ^61^, ^62^, ^63^, ^64^, ^65^, ^66^, ^67^, ^68^, ^69^, ^70^, ^71^, ^72^, ^73^, ^74^, ^75^, ^76^ Details of these studies are presented in the Supplemental materials (Supplemental Tables S7 and S8). The sex of humans, animals, cells, and tissues involved are documented in Supplemental Table S7 (under the column labelled “cell tissue”) and Supplemental Table S8 (under the column labelled “animal model”). This information was frequently difficult to ascertain in pre-clinical studies, especially concerning the in-vitro studies.

**Fig. 3:**
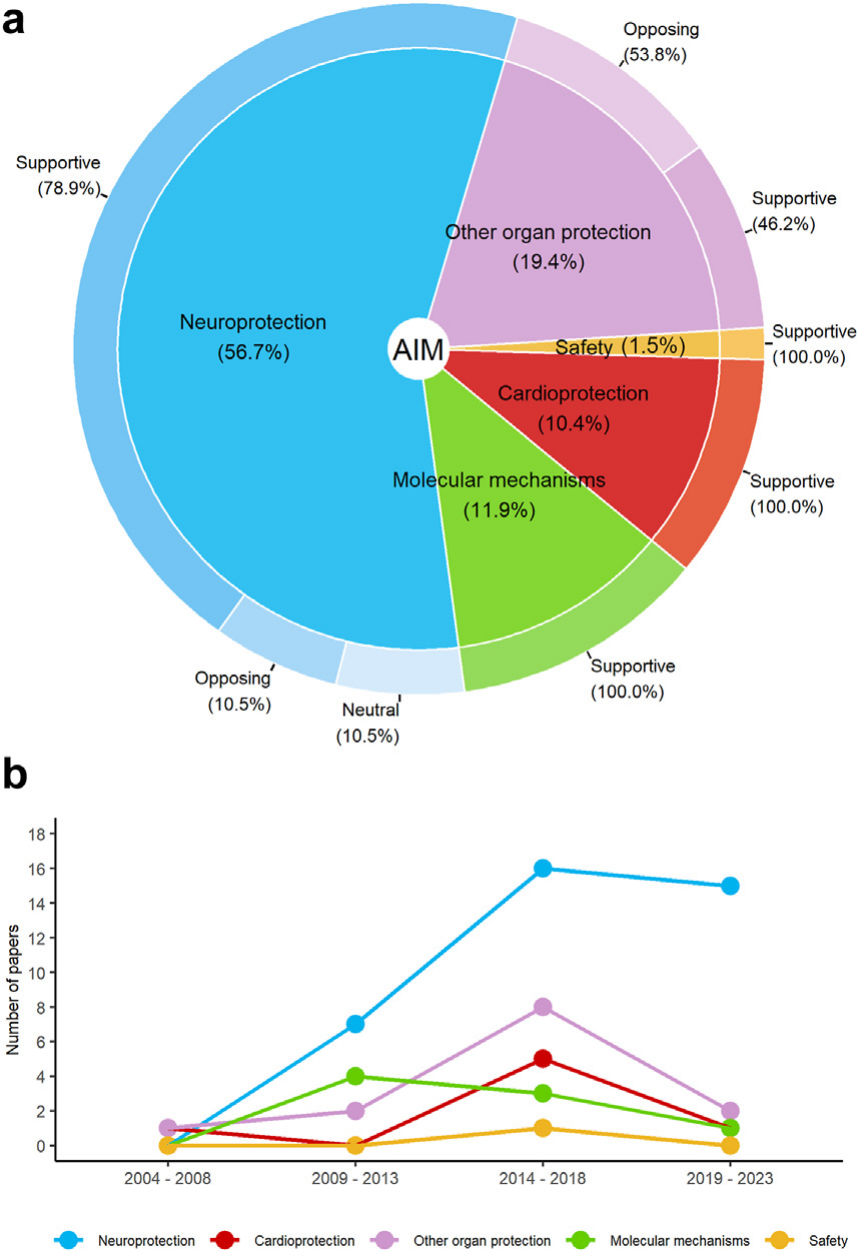
**Analysing the evolution of scientific objectives and published findings on the effects of Ar administration in the pre-clinical studies (*in vitro, ex vivo, in vivo*).** Data are referred to 60 pre-clinical studies (16 *in vitro*, 7 *ex vivo*, 31 *in vivo*, 6 with both *in vitro* and *in vivo*). **Panel a**. Distribution of primary aims regarding Ar administration effects: supportive, opposing, and neutral findings. “Supportive” means that the authors of the study found that Ar administration led to an improved condition in all the evaluated outcomes compared to the control group; “opposing” means that the authors of the study found that Ar administration did not find any benefit associated with Ar administration in any of the analysed outcomes; “neutral” means that the authors of the study found Ar benefits in some of the analysed outcomes and no benefits in the remaining. Of note, all the studies were positive regarding the safety of Ar administration. **Panel b.** Number of studies on Ar effect according publication year and investigated primary outcome on the effects of Ar administration.

Examining the pre-clinical studies from a temporal perspective, a clear evolution in the primary outcome over the years became evident (Fig. 3, panel b). Specifically, in the early 2000s most of the pre-clinical studies aimed to clarify Ar’s mechanisms of action and to assess its safety. Instead, starting from 2013, organ protection emerged as a prominent research theme with neuroprotection representing the field of interest most frequently investigated.

Ar neuroprotective effect after different types of injury was selected as the primary outcome in most of the pre-clinical studies (Fig. 3, panel a). Some studies investigated Ar effects on organ or tissue preservation and regeneration (i.e., kidney, liver, or heart),^33^, ^34^, ^35^, ^36^, ^37^, ^38^, ^39^^,^^49^^,^^59^ together with its molecular mechanisms of action and/or safety evidence during administration.^49^

The majority of the selected studies presented supporting evidence for the use of Ar, particularly in the context of neuroprotection, despite the variation in animal models, types of diseases, or treatment protocols (Fig. 3, panel a).^18^, ^19^, ^20^^,^^22^, ^23^, ^24^, ^25^, ^26^, ^27^^,^^29^, ^30^, ^31^, ^32^^,^^40^^,^^41^^,^^43^, ^44^, ^45^^,^^47^, ^48^, ^49^, ^50^, ^51^, ^52^, ^53^, ^54^, ^55^, ^56^, ^57^^,^^60^^,^^62^, ^63^, ^64^^,^^67^, ^68^, ^69^^,^^71^^,^^73^, ^74^, ^75^, ^76^

Nonetheless, a few studies reported non-beneficial or inefficient effects of Ar.^1^^,^^21^^,^^28^^,^^42^^,^^46^^,^^58^^,^^59^^,^^61^^,^^65^^,^^66^^,^^70^^,^^72^

Importantly, no significant adverse events have been documented in any study.

### Summary of evidence for *in vitro* studies

Twenty-two articles explored Ar effects *in vitro* settings (Supplemental Table S7).^18^, ^19^, ^20^, ^21^, ^22^, ^23^, ^24^, ^25^, ^26^, ^27^, ^28^, ^29^, ^30^, ^31^, ^32^^,^^42^^,^^52^, ^53^, ^54^, ^55^^,^^60^^,^^68^

Studies were predominantly performed on rodents’ neurons and hippocampal brain slices or on human cells (i.e., neuroblastoma, kidney, osteosarcoma, epithelium). Other three studies were conducted in rodent cardiomyocytes and one of this also included an additional analysis on human right atrial appendages,^60^ obtained from patients undergoing coronary artery bypass or valve replacement surgery. Lastly, one study was conducted on whole blood from rodents.^24^

The vast majority of the studies (18/22) investigated the protective effects of Ar on cell injury induced by different techniques: mechanical trauma (*in vitro* traumatic brain injury-TBI),^23^^,^^28^ oxygen-glucose-deprivation (OGD),^19^, ^20^, ^21^^,^^29^, ^30^, ^31^^,^^42^^,^^54^^,^^55^ and global metabolic stress (i.e., hypoxia or by drugs such as rotenone, STS, methotrexate, antimycin A, menadione, cisplatin and gentamicin).^18^^,^^19^^,^^25^^,^^27^^,^^32^^,^^52^^,^^53^^,^^60^^,^^68^ Ar neuroprotective effect was investigated in eight studies assessing distinct outcomes (i.e., cell viability, trauma intensity) with different techniques.^19^^,^^20^^,^^23^^,^^28^^,^^30^^,^^54^^,^^55^^,^^68^ The remaining studies (4/22) examined Ar molecular mechanisms, specifically investigating its activity on modulation of Extracellular signal-regulated kinases 1 and 2 signalling and Toll-like receptors (TLR-2 and TLR-4).^22^, ^23^, ^24^^,^^28^

In these studies, Ar incubation was set with a concentration varying from 25% to 95%, in air and/or O_2_ and with an exposure time varying from 5 min in a model of hypoxia in cardiomyocytes,^60^ to 72 h in a model of OGD condition.^20^

Overall, *in vitro* studies showed a widespread improvement in cell viability and survival, independently from the cells/tissue model, type of disease and Ar treatment protocol. Specifically, Ar reduced apoptosis via TLRs.^22^^,^^23^^,^^28^^,^^48^^,^^49^^,^^65^

Five out of twenty-two studies employed Ar administration as a preconditioning intervention,^21^^,^^26^^,^^27^^,^^31^^,^^32^ while two studies as a post conditioning one.^60^^,^^68^ Specifically, Ar preconditioning appeared effective in protecting against neuronal cell apoptosis, as well as inhibiting radical oxygen species induced oxidative stress in rat cardiomyocytes.^31^^,^^32^

Finally, in rat’s whole-blood Ar was shown to play effect on tissue plasminogen activator (tPA) enzymatic and thrombolytic efficacy, with a concentration-dependent dual effect.^42^ Indeed, low (25%) and high (75%) Ar concentrations block and increase tPA enzymatic and thrombolytic efficiency, respectively.

### Summary of evidence for *ex-vivo* studies

Seven *ex vivo* studies evaluating Ar efficacy, mainly on kidneys and lungs, were found (Supplemental Table S8).^33^, ^34^, ^35^, ^36^, ^37^, ^38^, ^39^ Most of them showed no benefit in terms of organ protection. In contrast, Kiss and colleagues, showed that preconditioning with Ar enhanced cardiac functional recovery in rat hearts arrested with histidine–tryptophan–ketoglutarate cardioplegia, thereby representing a potential cardioprotective approach to be used in cardiac surgery.^39^

### Summary of evidence for *in vivo* studies

A total of thirty-seven *in vivo* studies investigating Ar as potential protective agent for different organs (brain, heart, kidney, and liver) were found (Supplemental Table S8).^1^^,^^40^, ^41^, ^42^, ^43^, ^44^, ^45^, ^46^, ^47^, ^48^, ^49^, ^50^, ^51^^,^^53^, ^54^, ^55^, ^56^, ^57^, ^58^, ^59^, ^60^, ^61^, ^62^, ^63^, ^64^, ^65^, ^66^, ^67^, ^68^, ^69^, ^70^, ^71^, ^72^, ^73^, ^74^, ^75^, ^76^

**Fig. 4:**
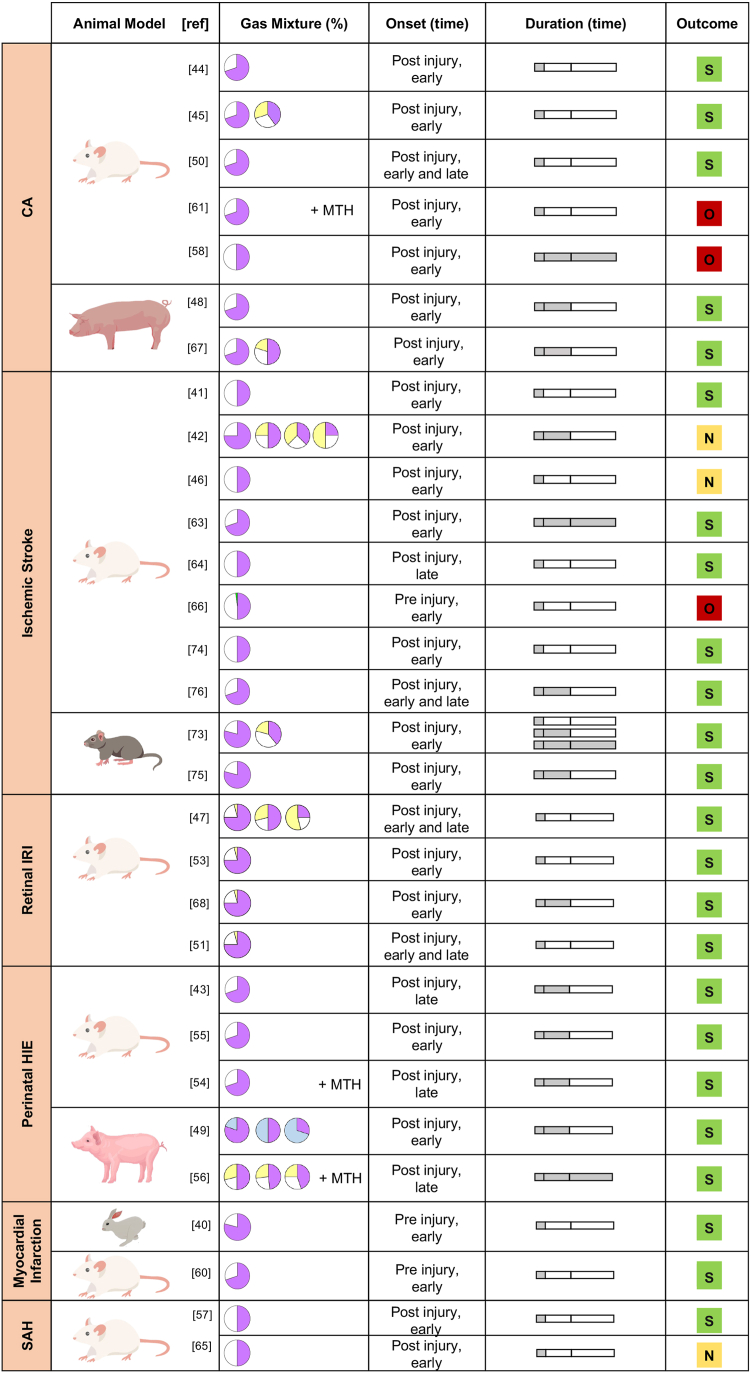
**Treatment protocols of all clinical conditions derived from ischemia reperfusion injury (CA, ischemic stroke, retinal IRI, perinatal HIE, myocardial infarction, SAH). Gas mixture.** The pie chart represents the blend composition, in violet the percentage of Ar, in white the percentage of oxygen, in yellow the percentage of nitrogen. **Onset timing.** Defining temporal parameters: “Early” within one and a half hours pre- or post-injury; “Late” after one and a half hours pre- or post-injury. **Duration (time) of administration.** The bar chart consists of three segments: the first represents durations of 1 h or less, the second represents durations between 1.5 h and 12 h (inclusive), and the third represents durations between 12 h and 24 h (inclusive). **Outcome**. The coloured square denotes the study outcome. “Supportive” (green) means that the authors of the study found that Ar administration led to an improved condition in all the evaluated outcomes compared to the control group; “opposing” (red) means that the authors of the study found that Ar administration did not find any benefit associated with Ar administration in any of the analysed outcomes; “neutral” (yellow) means that the authors of the study found Ar benefits in some of the analysed outcomes and no benefits in the remaining. Of note, all the studies were positive regarding the safety of Ar administration.

**Fig. 5:**
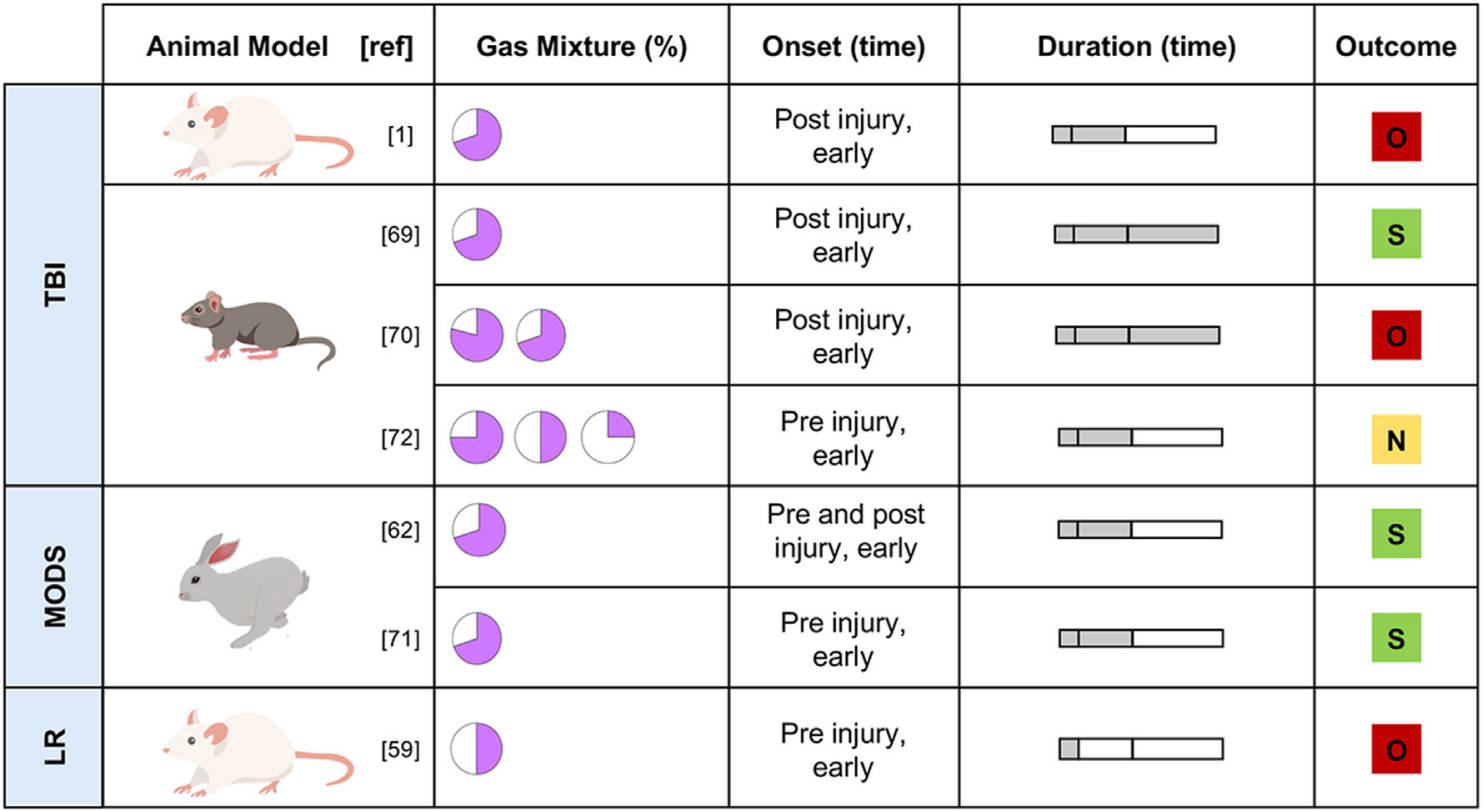
**Treatment protocols of all clinical conditions not derived from an IRI. Gas mixture.** The pie chart represents the blend composition, in violet the percentage of Ar, in white the percentage of oxygen, in yellow the percentage of nitrogen. **Onset timing.** Defining temporal parameters: “Early” within one and a half hours pre- or post-injury; “Late” after one and a half hours pre- or post-injury. **Duration (time) of administration.** The bar chart consists of three segments: the first represents durations of 1 h or less, the second represents durations between 1.5 h and 12 h (inclusive), and the third represents durations between 12 h and 24 h (inclusive). **Outcome**. The coloured square denotes the study outcome. “Supportive” (green) means that the authors of the study found that Ar administration led to an improved condition in all the evaluated outcomes compared to the control group; “opposing” (red) means that the authors of the study found that Ar administration did not find any benefit associated with Ar administration in any of the analysed outcomes; “neutral” (yellow) means that the authors of the study found Ar benefits in some of the analysed outcomes and no benefits in the remaining. Of note, all the studies were positive regarding the safety of Ar administration.

The majority of the studies (33/37) was conducted on rodent’s models (rats, rabbits, and mice),^1^^,^^40^, ^41^, ^42^, ^43^, ^44^, ^45^, ^46^, ^47^^,^^50^^,^^51^^,^^53^, ^54^, ^55^^,^^57^, ^58^, ^59^, ^60^, ^61^, ^62^, ^63^, ^64^, ^65^, ^66^^,^^68^, ^69^, ^70^, ^71^, ^72^, ^73^, ^74^, ^75^, ^76^ while others used swine models (4/37).^48^^,^^49^^,^^56^^,^^67^ A comprehensive collection of all different treatment protocols (dose, onset timing, duration of administration, outcome) related to a specific clinical condition and animal model is reported in Fig. 4, Fig. 5. Twenty-seven out of 37 articles provided supporting evidence on organ protection by Ar, four were neutral, while six were opposing. Available data suggested a pivotal role of Ar in CA, perinatal HIE, retinal IRI, ischemic stroke, SAH, myocardial infarction, TBI, and multiorgan dysfunction syndrome (MODS). Due to the variety of the reported diseases, two macro areas were identified: 1. Primary IRI-derived models: ischemic stroke, SAH, retinal IRI, perinatal HIE, myocardial infarction and CA (Fig. 4), and 2. Not primary IRI-derived models: TBI, MODS and a model of liver regeneration (LR) (Fig. 5).

The primary IRI-derived group represents the largest portion of the pre-clinical studies (30/37) conducted to evaluate the safety and efficacy of Ar administration as organ protectant.^40^, ^41^, ^42^, ^43^, ^44^, ^45^, ^46^, ^47^, ^48^, ^49^, ^50^, ^51^^,^^53^, ^54^, ^55^, ^56^, ^57^, ^58^^,^^60^^,^^61^^,^^64^^,^^66^, ^67^, ^68^^,^^73^, ^74^, ^75^, ^76^

Indeed, IRI plays a crucial role in the pathophysiology of multiple diseases and is commonly detected in different clinical conditions.^77^ These studies, mainly conducted in the last two decades, despite the use of different treatment protocols and multiple animal models, provided predominantly supportive evidence for Ar administration.

Only a few studies (7/37) were conducted on models not primary derived from IRI.^1^^,^^59^^,^^62^^,^^69^, ^70^, ^71^, ^72^ Experimental evidence regarding the neuroprotective effect of Ar on TBI provided mixed results, showing both positive and negative effects.^1^^,^^69^^,^^70^^,^^72^ Two studies, performed in a rabbit model of supra-coelic aortic cross clamping, focused on Ar organ protective properties as a potential therapeutic treatment for the MODS, reporting beneficial effects.^62^^,^^71^

### Neuroprotective effect of Ar administration

Given the significance of this topic, the neuroprotective effects of Ar administration are presented separately for each of the following four clinical conditions:

1. Cardiac arrest.

The use of Ar ventilation as a neuroprotective agent after CA has been explored in seven studies.^44^^,^^45^^,^^48^^,^^50^^,^^58^^,^^61^^,^^67^

In a rat model it has been showed that post–CA ventilation with an Ar/O_2_ 70/30 mixture for 1 h was associated with improved functional (Neurological Deficit Score, NDS; Open Field Test, OFT) and histological (neurodegeneration) outcomes.^44^^,^^45^^,^^50^ A dose dependent neuroprotective effect of Ar was also demonstrated. Surprisingly, Ar administration combined with therapeutic hypothermia (33 °C for 6 h) was instead not associated with any improvement in neurological recovery and neuronal damage.^61^

Subsequent studies in a CA swine model, testing different duration of no-flow time (8 and 12 min of untreated CA) and different concentrations of Ar (Ar/O_2_ 70/30 and Ar/N_2_/O_2_ 50/20/30), confirmed the dose-dependent neuroprotective effect of the noble gas in enhancing neurological functional recovery (NDS, and Neurologic Alertness Score, NAS), circulating biomarkers (Neuron Specific Enolase) and histopathological analyses.^48^^,^^67^

Lastly, a recent study testing the neuroprotective effect of an Ar/O_2_ 50/50 ventilation in a rat model of CA did not show any benefit.^58^

2. Perinatal hypoxic-ischemic encephalopathy.

All studies focusing on perinatal HIE showed the beneficial role of Ar,^54^, ^55^, ^56^ independently from the animal model and the treatment protocol. Moreover, Ar combined with hypothermia (33 °C and 35 °C), further reduced infarct volume,^54^^,^^55^ improved brain metabolism on magnetic resonance spectroscopy,^56^ provided a faster electroencephalogram recovery, and reduced cell death on histopathological analysis.^56^

3. Traumatic brain injury.

Four *in vivo* studies tested Ar ventilation on TBI,^1^^,^^69^^,^^70^^,^^72^ but only one confirmed the neuroprotective effect previously detected in the *in vitro* studies. Specifically, Moro et al.^69^ showed that a 24-h Ar/O_2_ 70/30 administration in TBI mice was associated with reduced vasogenic oedema and inflammatory markers, and improved cognitive deficits and sensorimotor recovery (Neuroscore, Simple Neuroassessment of Asymmetric Impairment, and Beam Walk Test (BWT)). The other studies, all conducted in rodents and using different treatment protocols, did not provide any differences in behavioural and neurological deficits nor in the severity of the brain injury.^1^^,^^67^^,^^69^

4. Stroke models: ischemic stroke, retinal ischemia, and reperfusion injury, and subarachnoid haemorrhage.

Ischemic stroke is the most investigated clinical condition for Ar treatment, with ten studies,^41^^,^^42^^,^^46^^,^^63^^,^^64^^,^^73^, ^74^, ^75^, ^76^ most of them performed through the occlusion of the middle cerebral artery in rodents. Only one study used photochemical technique to induce thrombosis of cerebral cortex vessels.

The initial studies demonstrated that Ar/O_2_ 50/50 inhalation was associated with decreased infarct volume, cortical neuroprotection, and better composite behavioural outcomes.^41^^,^^42^^,^^46^

More recently, additional data on neuroprotection have been collected, showing that Ar exhibits a pivotal role in reducing neuroinflammation and mitigating neurological deficits, despite the different treatment protocols used.^63^^,^^64^^,^^73^, ^74^, ^75^, ^76^

Furthermore, an ischemia-reperfusion model of ocular hypertension has been investigated in four studies, which provided all supportive data on Ar neuroprotection, i.e., reduction of both retinal ganglion cells damage and neuroinflammation, and its molecular mechanism.^47^^,^^51^^,^^53^^,^^68^

Finally, two studies investigated Ar effect on SAH reporting a decreased risk of mortality and a marked benefit on microglial inflammatory response and neuronal survival.^57^^,^^65^

### Other organ protective effects of Ar administration

The organ protective effect of Ar has been examined across various organs and clinical conditions: two recent studies have demonstrated Ar organ protective effect in a rabbit model of MODS.^62^^,^^71^

In both studies, Ar attenuated clinical and biological changes on cardiovascular, renal, and gut systems.

On the context of organ regeneration, a rat model of IRI following partial hepatectomy, provided negative results, revealing that Ar may cause delayed regeneration although it did not completely abolish the repair mechanisms.^59^

Cardioprotection was then investigated in a few studies, either as a primary or secondary outcome. In a rat model of myocardial infarction, Ar seemed to have strong cardioprotective properties when administered as post-conditioning intervention, thus highlighting the possible therapeutic role in this condition.^60^ Additional evidence derives from studies in a model of myocardial infarction and CA in a swine model, where Ar led to improved left ventricular ejection fraction and decreased high-sensitivity cardiac troponin T release, when compared to the control treatment.^67^

### Safety of Ar administration

Most of the *in vivo* studies (16/37) assessed and confirmed safety of Ar administration.^41^^,^^42^^,^^44^, ^45^, ^46^^,^^48^, ^49^, ^50^^,^^61^^,^^62^^,^^64^^,^^67^^,^^71^^,^^72^^,^^74^^,^^76^

Specifically, studies on CA swine models consistently revealed that Ar ventilation had no detrimental effects on both haemodynamics and respiratory gas exchange. A recent study on a rabbit model of MODS further confirmed the absence of haemodynamic impairment during Ar administration.^62^

### Meta-analysis on neuroprotective effect of Ar administration

Six *in vitro* and nineteen *in vivo* studies provided data for at least one of the investigated outcomes.^1^^,^^19^^,^^20^^,^^23^^,^^28^^,^^30^^,^^41^, ^42^, ^43^, ^44^, ^45^, ^46^^,^^48^^,^^50^^,^^55^^,^^57^^,^^58^^,^^64^^,^^65^^,^^67^^,^^69^^,^^70^^,^^74^^,^^75^

The risk of bias assessment for all included studies is reported in Supplemental Table S9 (*in vivo* studies) and in Supplemental Table S10 (*in vitro* studies).

For *in vivo* studies, the SYRCLE’s risk of bias tool assessment showed scores ranging between four to seven.^12^ Seven *in vivo* studies showed a low risk of bias in three or four out of ten potential sources of bias (Supplemental Table S9, total score equalling four or less).^1^^,^^42^^,^^43^^,^^46^^,^^55^^,^^65^^,^^75^ Due to inadequate information in the publications, evaluating performance bias (bias associated with blinding of participants and personnel) and random outcome assessment was not feasible for all studies. With regard to the blinding of outcome assessment, adherence was observed in nearly all studies (15 out of 19), with the exception of four.^1^^,^^42^^,^^43^^,^^65^ All publications showed a low risk of reporting bias (selective inclusion of outcomes in the publication of the study on the basis of the results), while the risk of selection bias was high for 11 studies.^1^^,^^42^^,^^43^^,^^46^^,^^48^^,^^55^^,^^65^^,^^67^^,^^69^^,^^70^^,^^75^

Regarding the quality of the included *in vitro* studies,^19^^,^^20^^,^^23^^,^^28^^,^^30^^,^^55^ OHAT risk of bias rating tool showed that the majority of studies demonstrated intermediate or high quality across all domains considered for bias assessment (Supplemental Table S10). The item with the highest risk of bias was “selection bias”, due to the nature of laboratory experiment. Of note, attrition and confounding biases were unreported in the publications of all *in vitro* studies.

The results of the meta-analysis are presented with a distinction between *in vitro* studies, underlining cell protection, and *in vivo* studies, which are divided into the functional, physiological, and histological domains.

#### *In vitro* studies—cell protection

Out of eight *in vitro* studies exploring the neuroprotective effect of Ar, seven reported cell protection indexes (cell viability and trauma intensity) and were deemed eligible for inclusion in the meta-analysis.^19^^,^^20^^,^^23^^,^^28^^,^^30^^,^^54^^,^^55^ Among these, Harris K. et al.^23^ reported cell protection indexes at various timepoints post-trauma (24, 48, and 72 h) and the meta-analysis only included data observed after 72 h for the sake of comparability with other *in vitro* studies using a traumatic injury model.^20^^,^^28^ Moreover, the study conducted by Zhao et al.^54^ was excluded because it involved the use of Ar in combination with mild therapeutic hypothermia.

**Fig. 6:**
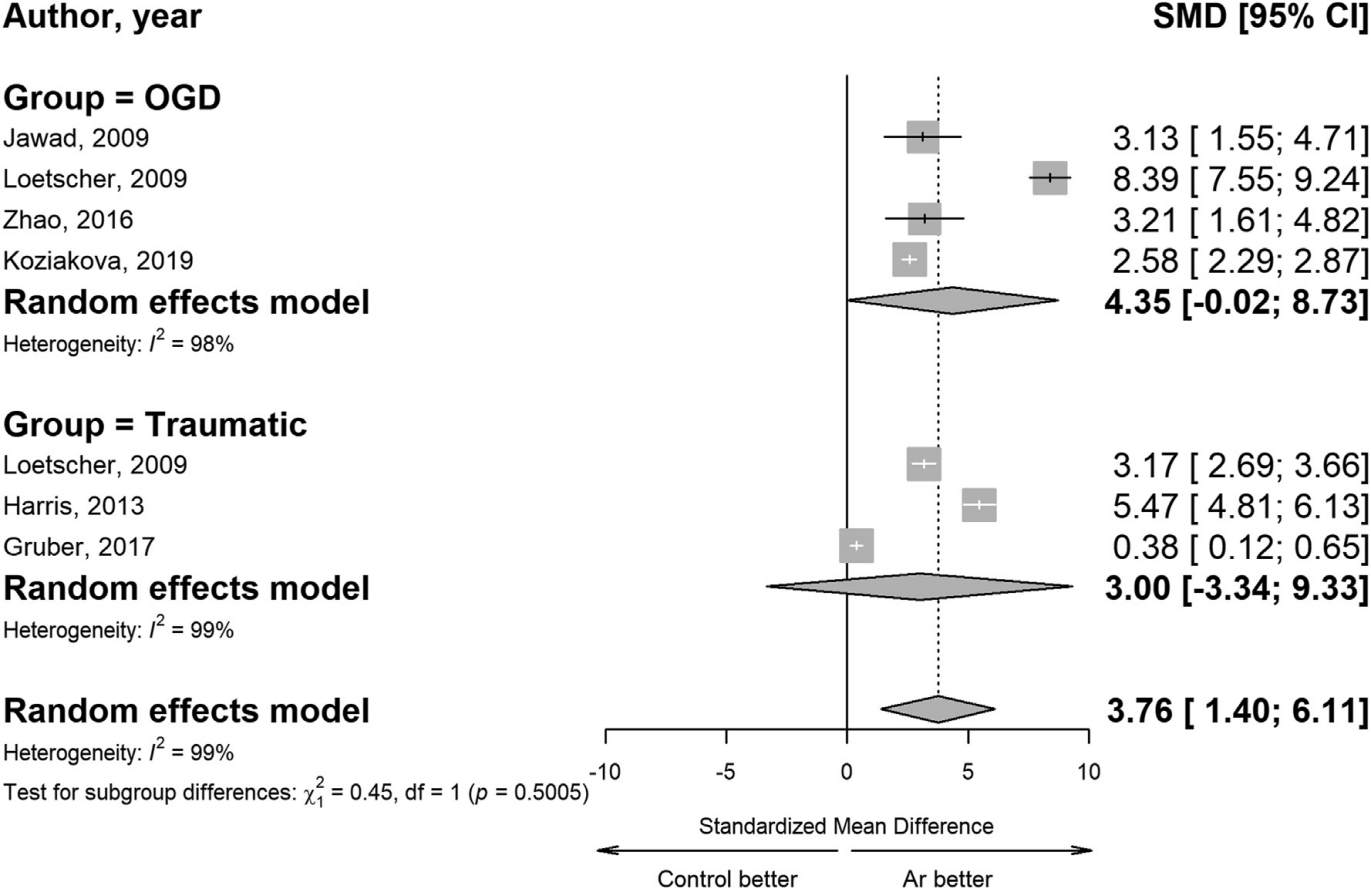
**Forest plot of Ar administration effect on cell protection *in vitro* studies.** Random effects models were used to estimate the pooled estimates for cell protection both overall and by subgroups injury models (OGD and traumatic brain injury). Data are expressed as SMD and 95% CI. Size of each square represents the study weight in the analysis. The diamond represents the pooled effect from the included studies (the width of the diamond represents the 95% CI for the overall effect) both overall and by subgroups injury models. CIs crossing zero (vertical line) indicate inconclusive results regarding the support for or against Ar.

Overall, the pooled effect estimate showed a neuroprotective effect of Ar *in vitro* experiments with a SMD of 3.76 (95% CI 1.40–6.11) (Fig. 6). A subgroup meta-analysis, stratified by the technique for inducing cell injury (OGD, traumatic), did not reveal any significant differences in the neuroprotective effect of Ar (test for subgroup differences, p = 0.5005). Notably, considerable heterogeneity was observed both across all studies and within subgroups, with the I^2^ statistic exceeding 90%, also visually detectable in the funnel plot (Supplemental Fig. S1, panel a).

#### *In vivo* studies—functional domain

Six randomised animal trials investigated the neurological outcome, which was assessed through the NDS measured from 24 h after the end of Ar administration till four days later.^44^^,^^45^^,^^48^^,^^50^^,^^58^^,^^67^

Five studies evaluated cognitive and locomotor outcome using several tests (Morris Water Maze Test, OFT, Tape removal test, Vertical pole test, Rotarod test, Barnes maze test, and BWT), over the same time interval reported above (24 h–4 days after treatment).^44^^,^^45^^,^^57^^,^^69^^,^^70^

**Fig. 7:**
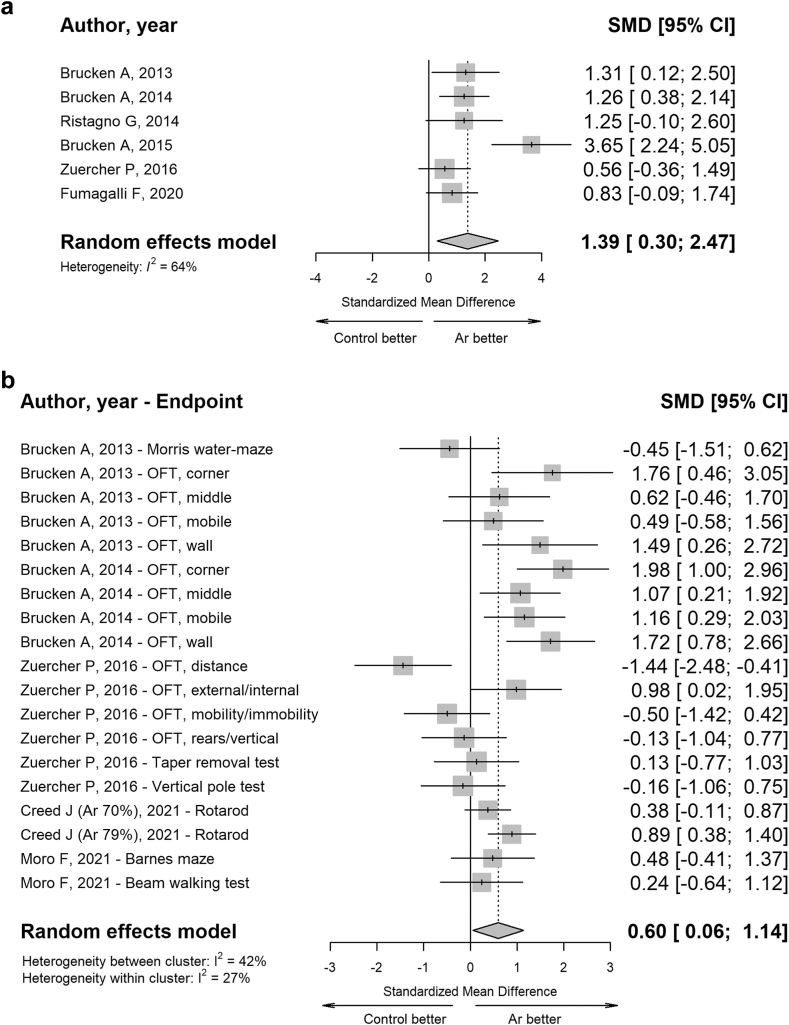
**Forest plots of Ar administration effects on functional domain in pre-clinical studies (*in vivo*).** Random effects models were used to estimate the pooled estimates for neurological outcome (**panel a**). Three-level nested random effects model was used to estimate the pooled estimates for cognitive and locomotor outcome (**panel b**). Data are expressed as SMD and 95% CI. Size of each square represents the study weight in the analysis. The diamond represents the overall pooled effect from the included studies (the width of the diamond represents the 95% CI for the overall effect). CIs crossing zero (vertical line) indicate inconclusive results regarding the support for or against Ar.

The meta-analysis indicates that the Ar treatment exhibited favourable results in NDS (SMD 1.39, 95% CI: 0.30–2.47) and in cognitive and locomotor tests (SMD 0.60, 95% CI: 0.06–0.14) (Fig. 7, panel a–b).

A high level of heterogeneity between studies was not detected for either outcome (I^2^ < 75%).

Subgroup meta-analysis revealed a statistically significant difference in the neuroprotective effect of Ar on neurological outcome based on Ar concentration (test for subgroup difference, p = 0.0115): a pooled effect size of 1.85 (95%CI 0.52–3.19) was observed when the Ar concentration reached 70%, whereas a value of 0.63 (95%CI 0.38–0.87) was noted when the Ar concentration was less than 70% (Supplemental Fig. S2). No statistically significant difference between groups was observed when employing cognitive and locomotor tests (Supplemental Fig. S3).

Funnel plots were reported in Supplemental Fig. S1 (panel b–c), revealing a potential risk of reporting bias for neurological outcome.

#### *In vivo* studies—physiological domain

In the meta-analysis, nine randomised animal trials were included as they reported haemodynamic, metabolic, and respiratory parameters evaluated immediately after the end of treatment or, at most, within 24 h.^41^^,^^42^^,^^44^^,^^45^^,^^48^^,^^50^^,^^64^^,^^67^^,^^74^

**Fig. 8:**
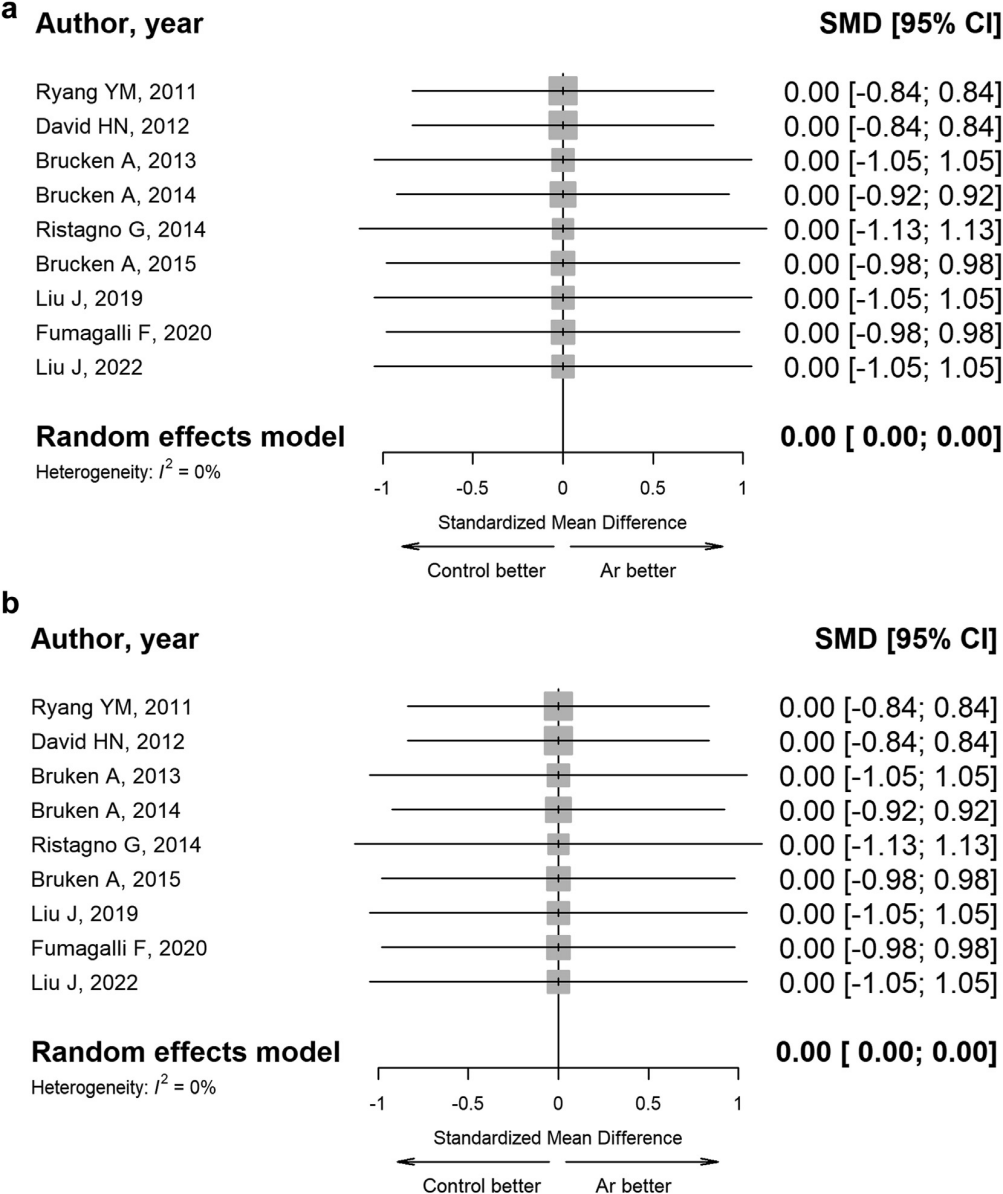
**Forest plots of Ar administration effects on respiratory exchange outcome in pre-clinical studies (*in vivo*).** Random effects models were used to estimate the pooled estimates for gas exchange parameters, such as arterial partial pressure of oxygen (**panel a**) and arterial partial pressure of carbon dioxide (**panel b**). Data are expressed as SMD and 95% CI. Size of each square represents the study weight in the analysis. The diamond represents the overall pooled effect from the included studies (the width of the diamond represents the 95% CI for the overall effect). CIs crossing zero (vertical line) indicate inconclusive results regarding the support for or against Ar.

For all endpoints, pooled effect sizes did not favour or oppose the utilisation of Ar (Fig. 8, panel a–b, and Supplemental Table S11).

#### *In vivo* studies—histological domain

At the conclusion of the experiment, histological assessments were conducted in 14 randomised animal trials.^1^^,^^43^^,^^46^^,^^48^^,^^55^^,^^57^^,^^58^^,^^64^^,^^65^^,^^67^^,^^69^^,^^70^^,^^74^^,^^75^

**Fig. 9:**
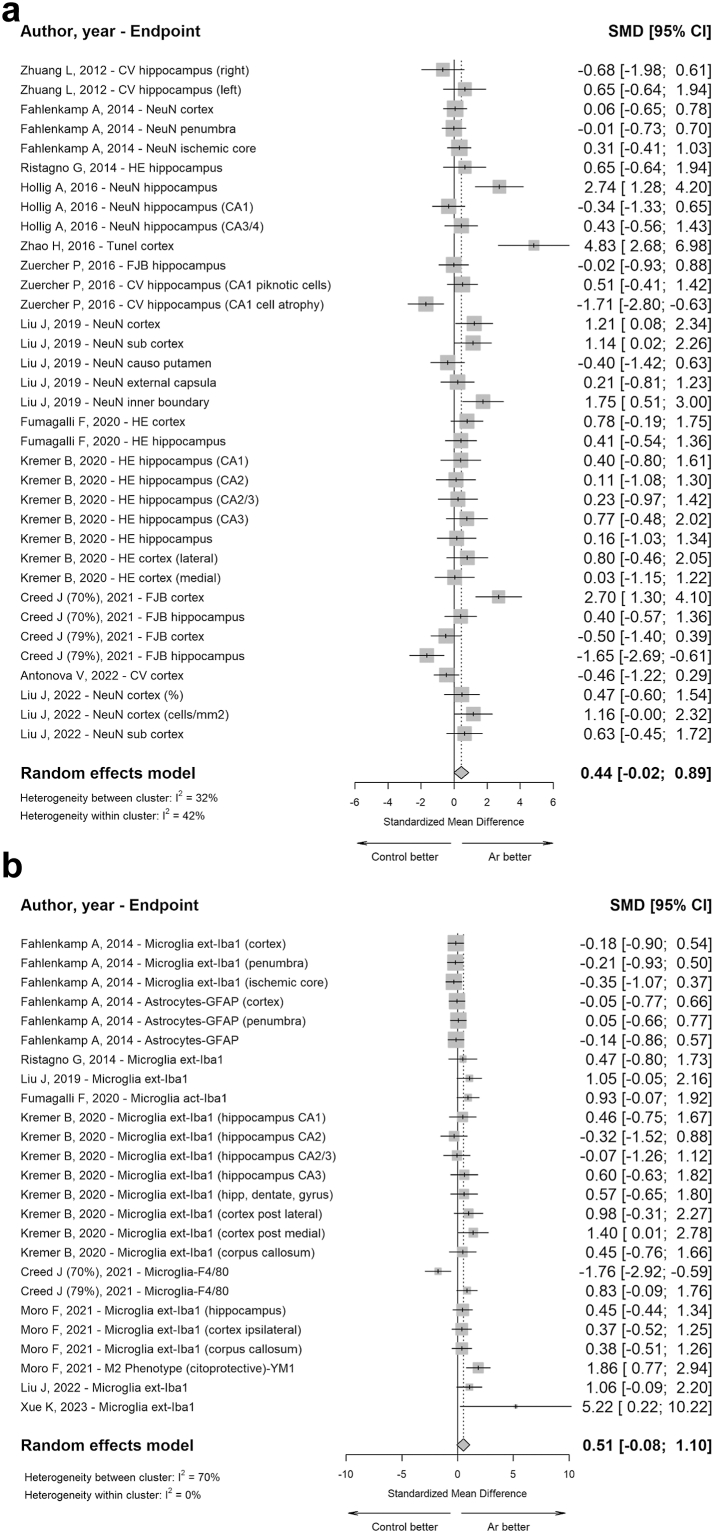
**Forest plots of Ar administration effects on histological outcomes in pre-clinical studies (*in vivo*).** Three-level nested random effects model was used to estimate the pooled estimates for neurodegeneration (**panel a**) and inflammation (**panel b**) outcome. Data are expressed as SMD and 95% CI. Size of each square represents the study weight in the analysis. The diamond represents the overall pooled effect from the included studies (the width of the diamond represents the 95% CI for the overall effect). CIs crossing zero (vertical line) indicate inconclusive results regarding the support for or against Ar.

Regarding the neurodegeneration outcome, the meta-analysis estimated a pooled effect size of 0.44 (95%CI: −0.02 to 0.89) with low heterogeneity between and within clusters (I^2^ at 32% and 42%, respectively) (Fig. 9, panel a). Funnel plot (Supplemental Fig. S1, panel m) revealing a potential risk of reporting bias (test for asymmetry, p < 0.0001).

Outliers were observed in five effect sizes (three positive effect sizes for Ar utilisation, two negative effect size for no Ar utilisation) originating from three studies with a mediocre quality score (between five and seven).^57^^,^^58^^,^^70^ After their removal from the model, the overall effect size decreased to 0.27 (95% CI: 0.05–0.50), favouring Ar utilisation (Supplemental Fig. S3, panel a).

Out of the seven *in vivo* studies with a quality score of four or lower, five assessed the neurodegeneration outcome.^1^^,^^43^^,^^46^^,^^55^^,^^65^ The exclusion of these studies from the meta-analysis showed an overall effect size of 0.44 (95%CI: −0.12 to 1.00) with a decrement in heterogeneity between studies (I^2^ = 15%) (Supplemental Table S12).

When considering measures obtained from the hippocampus, the overall effect size was 0.14 (95% CI: −0.34 to 0.63), and it remained statistically not significant even after the removal of outlier effect sizes (SMD 0.26, 95% CI: −0.06 to 0.57) or low quality studies (SMD 0.10, 95%CI −0.75 to 0.95) (Supplemental Table S13). Similar findings were obtained when measures on cortex were analysed (Supplemental Table S13).

In relation to the inflammation outcome, the meta-analysis revealed an overall effect size of 0.51 (95% CI: −0.08 to 1.10), indicating no statistical favour toward Ar utilisation (Fig. 9, panel b). Funnel plot (Supplemental Fig. S1, panel n) revealing a potential risk of reporting bias (test for asymmetry, p = 0.0061).

Three *in vivo* studies with a quality score of four or lower assessed the inflammation outcome.^46^^,^^65^^,^^75^ Excluding these studies, the overall effect size was 0.69 (95%CI −0.17 to 1.54) (Supplemental Table S14). In addition, removing two outlier effect sizes from two trials (one positive and one negative effect size),^70^^,^^75^ the overall effect size shifted to 0.54 (95% CI: 0.15–0.92), favouring Ar utilisation (Supplemental Table S14). When considering measures obtained from the hippocampus and cortex, the overall effect size was 0.22 (95% CI: −0.62 to 1.06) and 0.75 (95% CI: 0.03–1.46), respectively (Supplemental Table S15).

Additionally, inflammation outcome from the cortex area indicated a favourable trend toward Ar utilisation (SMD 1.04, 95%CI 0.01–2.09), excluding studies with low scores on the quality scale (Supplemental Table S15).^46^^,^^65^

Subgroup meta-analysis did not show any significant difference in Ar the neuroprotective effect on histological outcomes based on Ar concentration (Supplemental Figs. S4 and S5).

### Summary of evidence for clinical studies

The growing interest in potential clinical application led to a deeper investigation into the Ar inhalation in human physiology. Thus, Grune et al.,^17^ evaluated the effects of short-term inhalation (15 min) of 70% Ar in 30 patients undergoing elective coronary surgery. The authors did not find any changes in cerebral circulation, evaluated through mean blood flow velocity of the middle cerebral artery and cerebral perfusion pressure, and in cerebral metabolism, evaluated through arterio-jugular venous difference in glucose, lactate, and oxygen, thus confirming the drug safety.

## Discussion

The present study presents a comprehensive review and analysis of the existing literature to examine the evidence for the use of Ar in various clinical conditions. With the exception of one study, all available research is pre-clinical. Despite variations in animal models and treatment protocols, the collective findings consistently indicate the potential of Ar as a treatment for organ protection across multiple conditions. The results of the meta-analysis support the neuroprotective effect of Ar, demonstrating improvements in cell protection, in neurological, cognitive and locomotor outcomes, mitigation of neurodegeneration, and reduction of inflammation. Importantly, no adverse events are reported in haemodynamic, metabolic, and respiratory parameters, providing a compelling basis for translating Ar use into human applications.

The systematic review identified 61 studies investigating the role of Ar, comprising 16 *in vitro*,^18^, ^19^, ^20^, ^21^, ^22^, ^23^, ^24^, ^25^, ^26^, ^27^, ^28^, ^29^, ^30^, ^31^, ^32^^,^^52^ seven *ex vivo*,^33^, ^34^, ^35^, ^36^, ^37^, ^38^, ^39^ and 37 *in vivo* studies^1^^,^^40^, ^41^, ^42^, ^43^, ^44^, ^45^, ^46^, ^47^, ^48^, ^49^, ^50^, ^51^^,^^53^, ^54^, ^55^, ^56^, ^57^, ^58^, ^59^, ^60^, ^61^, ^62^, ^63^, ^64^, ^65^, ^66^, ^67^, ^68^, ^69^, ^70^, ^71^, ^72^, ^73^, ^74^, ^75^ (six of which also included *in vitro* sub-studies)^42^^,^^53^, ^54^, ^55^^,^^60^^,^^68^ and one clinical study.^17^ A substantial portion of the included studies supported the potential role of Ar as an organ protective agent, particularly in the context of neuroprotection, while highlighting the safety of its administration. A minority of studies did not show benefits associated with Ar administration. Differently from *in vivo* studies, most of the *ex vivo* studies did not show any benefit associated with Ar administration, probably due to the challenges in developing a suitable model that accurately reproduce the *in vivo* conditions. Similarly to findings by Antonova et al.,^1^ this review underscored significant heterogeneity in animal species and treatment protocols (Ar dose, onset timing, duration of the treatment), clearly emphasising the need of future studies to define optimal administration strategies for each specific clinical condition. Notably, CA was the clinical condition that exhibited lower heterogeneity in treatment protocol across the studies, supporting a clinical transition with a clinical trial currently underway (NCT05482945). Considering the effectiveness of Ar in organ protection demonstrated in pre-clinical models of conditions other than CA (i.e., ischemic stroke, TBI, SAH, perinatal HIE, MODS), the establishment of a precise and standardised treatment protocol may facilitate its clinical application also in these contexts.

The meta-analysis specifically focused on the neuroprotective effect of Ar and used data from *in vitro* and *in vivo* studies.^1^^,^^19^^,^^20^^,^^23^^,^^28^^,^^30^^,^^41^, ^42^, ^43^, ^44^, ^45^, ^46^^,^^48^^,^^50^^,^^55^^,^^57^^,^^58^^,^^64^^,^^65^^,^^67^^,^^69^^,^^70^^,^^74^^,^^75^

Ar demonstrated a significant neuroprotective effect *in vitro* experiments with no significant differences found in subgroup analysis based on cell injury induction techniques (OGD and traumatic brain injury). Nonetheless, high overall heterogeneity was observed across all studies and within the subgroups, suggesting that the overall estimate may not accurately reflect the real average treatment effect *in vitro* setting.

The *in vivo* meta-analysis summarised results from different neurological (NDS, NAS) and cognitive and locomotor tests (Morris water maze test, OFT, Tape removal test, Vertical pole test, Rotarod test, Barnes maze test, and BWT), demonstrating that Ar administration consistently enhanced functional outcomes in pre-clinical models of CA and TBI. Similar beneficial effects were observed in histological outcomes related to neurodegeneration and neuroinflammation. Overall, these findings support the use of Ar as a neuroprotective agent in different pre-clinical models of clinical conditions, such as CA, TBI, ischemic stroke, perinatal HIE, and SAH. In addition, an analysis of Ar effects on haemodynamic (mean arterial pressure, heart rate, cardiac output), metabolism (base excess, lactate, pH, glucose) and gas exchange (arterial partial pressure of oxygen, arterial partial pressure of carbon dioxide) revealed no adverse systemic events, confirming the safety of the treatment in animal models.

### Strengths and limitations

Despite high overall heterogeneity observed across all *in vitro* studies included in the meta-analysis evaluating the potential effects of Ar on cell protection, there is a consistent trend or effect noted across the included studies. This consistency strengthens the reliability of the findings and implies the robustness of the observed effect. Therefore, it may still be valuable and informative to present these findings. This comprehensive overview of available evidence contributes to a broader understanding of the topic, even in the presence of heterogeneity due to the different study designs (i.e., clinical condition, injury model, treatment protocol, and cell or tissue involved).

Concerning meta-analyses based on *in vivo* findings, the neuroprotective effects of Ar may be influenced by various aspects of the treatment protocol, including dose, onset timing and duration of administration. Due to the limited number of studies, a comprehensive joint analysis of these aspects was not feasible. Nevertheless, our exploratory subgroup meta-analysis revealed a statistically significant difference in the neuroprotective effect of Ar on neurological outcome based on its concentration. This analysis improves our understanding by providing additional insights and points out promising directions for future research activities on Ar effects, with a specific focus on the concentration.

Sensitivity analyses, based on the exclusion of low-quality studies and on the removal of single extreme effect sizes, have strengthened the robustness of the study findings. We highlight that the removal of individual extreme effect sizes was specifically applied to histological outcomes characterised by multiple measures, where potential sources of errors affecting outcome measurements, such as sampling, fixation, and sectioning errors of cells or tissues, could also be present. This approach prevented the entire study from being excluded from the meta-analysis, thereby mitigating the potential impact of publication bias on the overall estimates. While there is a chance we accidentally removed some reliable information, it is fundamental to mention that this did not affect the main conclusions of our meta-analysis, thereby highlighting the strength and reliability of our findings. The systematic review and meta-analysis of data derived from pre-clinical studies represent a crucial research domain addressing challenges in clinical translation of this treatment. This strategy proves essential in generating empirical evidence, fostering robust experimental design, guiding research strategy, and enhancing the support for funding applications. Nevertheless, this area is relatively recent and considerably less developed when compared with the structured approach developed for the clinical setting (Cochrane methodology). For example, the SYRCLE research group has made a significant contribution to the advancement and promotion of standardised approaches for systematic review and meta-analysis of pre-clinical data.^12^ However, one limitation of meta-analyses of pre-clinical studies relies in the heterogeneity of their study design, related to the choice of the animal species, the disease and injury model, the treatment protocol or the control group. Moreover, the quality of the studies may vary considerably. This can impact the estimate of the overall effect sizes. In our case, despite meticulous adherence to SYRCLE guidelines and the methodology outlined by Vesterinen HM et al.,^12^^,^^14^ conducting a subgroup analysis to investigate all sources of heterogeneity was not feasible. This limitation arose from the restricted number of studies available for each outcome of interest. Furthermore, adhering to Sterne et al.’s guidelines,^78^ the limited number of studies included for each outcome has hindered our ability to perform a statistical assessment of reporting bias using appropriate statistical tests (such as the Egger’s regression test) for all outcomes. Despite this limitation, this systematic review with meta-analysis on pre-clinical finings provides pooled results on several specific outcomes related to the neuroprotective effect of Ar, thus offering valuable evidence to guide research strategy and support the clinical translational potentials of Ar administration.

### Future implications

The extensive pre-clinical evidence outlined by the results of both the systematic review and the meta-analysis provides support for the clinical transition of Ar. Particularly, the meta-analysis supports neuroprotection as one of the most effective properties of Ar. Due to the lower heterogeneity in the Ar treatment protocols in pre-clinical models of CA compared with other clinical conditions, CA emerges as a candidate for the transition from experimental studies to human trials. Furthermore, it has been recently demonstrated, in a swine model of CA, the feasibility of administering an Ar/O_2_ 70/30 mixture for 4 h using a modified ventilator (Bellavista 1000, IMT Medical Buchs, Switzerland).^79^ The CardioPulmonary Resuscitation with Argon trial has recently started; it is an Italian multicentre, phase I/II, randomised, controlled, single blinded study aimed to evaluate safety and feasibility of an Ar/O_2_ 70/30 mixture in patients resuscitated from an out-of-hospital CA (NCT05482945). It is conceivable that these findings together with the refinement of the Ar administration protocols could serve as rationale and foundation for undertaking additional human studies. Clinical studies could extend beyond CA to explore the neuroprotective effects of Ar, potentially yielding significant benefits in patient outcomes for various conditions.

## Contributors

G.M.: Conceptualisation, investigation, data validation, visualisation, writing—original draft; G.F.: Conceptualisation, investigation, data validation, visualisation, writing—review & editing; F.M.: Conceptualisation, formal analysis, software, data validation, writing—review & editing; A.M.: Conceptualisation, data validation, writing–review & editing; I.S.: Visualisation, review & editing; F.F.: Visualisation, review & editing; M.C.: Visualisation, review & editing; F.Mo.: Visualisation, review & editing; D.D.G.: Visualisation, review & editing; M.P.: Supervision, review & editing; A.Z.: Supervision, review & editing; G.G.: Funding acquisition, methodology, review & editing; G.R.: Conceptualisation, funding acquisition, project administration, methodology, writing—review & editing. All authors read and approved the final version of the manuscript.

## Data sharing statement

The data collected for this study can be provided upon reasonable request to the corresponding author.

## Declaration of interests

G.G received funding from Fischer&Paykel, MSD, Pfizer, and received fees from Getinge, Draeger Medical, Cook, MundiPharma, Fischer&Paykel, Pfizer.

The remaining authors have disclosed that they do not have any potential conflicts of interest.
