# Supplementary material for: Treatment with inhaled Argon: a systematic review of pre-clinical and clinical studies with meta-analysis on neuroprotective effect
Source: eBioMedicine. 2024 Apr 30;103:105143. doi: 10.1016/j.ebiom.2024.105143 (PMC11070688; doi:10.1016/j.ebiom.2024.105143)
Supplement: Supplemental Figs. S1–S5 and Tables S1–S14 [file mmc1.docx]

**SUPPLEMENTAL DATA**

**TREATMENT WITH INHALED ARGON: A SYSTEMATIC REVIEW OF PRE-CLINICAL AND CLINICAL STUDIES WITH META-ANALYSIS ON NEUROPROTECTIVE EFFECT**

Giulia Merigo, Gaetano Florio, Fabiana Madotto, Aurora Magliocca, Ivan Silvestri, Francesca Fumagalli, Marianna Cerrato, Francesca Motta, Daria De Giorgio, Mauro Panigada, Alberto Zanella, Giacomo Grasselli, Giuseppe Ristagno

[Guideline and checklists for reporting systematic review 2](#_Toc161929705)

[Table S1. PRISMA 2020 item checklist 2](#_Toc161929706)

[Table S2. PRISMA 2020 for abstract checklist 5](#_Toc161929707)

[Search strategy for systematic review 6](#_Toc161929708)

[Table S3. Search algorithm 1 - Search for clinical and pre-clinical (in vivo and ex-vivo) studies 6](#_Toc161929709)

[Table S4. Search algorithm 2 - Search for in vitro studies 6](#_Toc161929710)

[Table S5. Combination of two search algorithms 6](#_Toc161929711)

[Details for outcomes included in the meta-analysis of neuroprotective effect of Ar in *in vivo* studies 7](#_Toc161929712)

[Table S6. Endpoints used for the assessment of Argon neuroprotective effect 7](#_Toc161929713)

[Characteristics of studies included in the systematic review and in the meta-analysis 8](#_Toc161929714)

[Table S7. Characteristics of in vitro studies included in the systematic review 8](#_Toc161929715)

[Table S8. Characteristics of in vivo and ex vivo studies included in the systematic review 11](#_Toc161929716)

[Table S9. Risk of bias summary for in vivo studies included in the meta-analysis 16](#_Toc161929717)

[Table S10. Risk of bias summary for in vitro studies included in the meta-analysis 17](#_Toc161929718)

[Additional findings from meta-analysis 18](#_Toc161929719)

[Table S11. Results of meta-analysis on haemodynamic and metabolic outcome (physiological domain) 18](#_Toc161929720)

[Table S12. Results of meta-analysis on neurodegeneration outcome, related to the histological domain 19](#_Toc161929721)

[Table S13. Results of meta-analysis on neurodegeneration outcome (histological domain) in hippocampus and cortex 20](#_Toc161929722)

[Table S14. Results of meta-analysis on neuroinflammation outcome, related to the histological domain 21](#_Toc161929723)

[Table S15. Results of meta-analysis on inflammatory outcome (histological domain) in hippocampus and cortex 22](#_Toc161929724)

[Figure S1. Funnel plots of Ar effect estimates from each study, according to the outcome. 23](#_Toc161929725)

[Figure S2. Forest plot of neurological outcome (functional domain) by percentage of Ar concentration (Ar < 70% and Ar ≥ 70%) 24](#_Toc161929726)

[Figure S3. Forest plot of cognitive and locomotor outcome (functional domain) by percentage of Ar concentration (Ar < 70% and Ar ≥ 70%) 25](#_Toc161929727)

[Figure S4. Forest plot of neurodegeneration outcome (histological domain) by percentage of Ar concentration (Ar < 70% and Ar ≥ 70%) 26](#_Toc161929728)

[Figure S5. Forest plot of neuroinflammation outcome (histological domain) by percentage of Ar concentration (Ar < 70% and Ar ≥ 70%) 27](#_Toc161929729)

[REFERENCES 28](#_Toc161929730)

**Guideline and checklists for reporting systematic review**

This systematic review adhered to the PRISMA 2020 guidelines. Detailed PRISMA 2020 checklists, including the abstract checklist, were provided in ***Table S1*** and ***Table S2***.

**Table S1. PRISMA 2020 item checklist**

| **Section and Topic** | **Item**  **#** | **Checklist item** | **Location where item is reported** |
| --- | --- | --- | --- |
| **TITLE** | | |  |
| Title | 1 | Identify the report as a systematic review. | Identified as a “Systematic Review and Meta-Analysis” |
| **ABSTRACT** | | |  |
| Abstract | 2 | See the PRISMA 2020 for Abstracts checklist (see ***Table S2***). | All items included. |
| **INTRODUCTION** | | |  |
| Rationale | 3 | Describe the rationale for the review in the context of existing knowledge. | See *Introduction* |
| Objectives | 4 | Provide an explicit statement of the objective(s) or question(s) the review addresses. | See *Introduction* |
| **METHODS** | | |  |
| Eligibility criteria | 5 | Specify the inclusion and exclusion criteria for the review and how studies were grouped for the syntheses. | See *Methods* (Search strategy and study selection). |
| Information sources | 6 | Specify all databases, registers, websites, organisations, reference lists and other sources searched or consulted to identify studies. Specify the date when each source was last searched or consulted. | See *Methods* (Search strategy and study selection). |
| Search strategy | 7 | Present the full search strategies for all databases, registers and websites, including any filters and limits used. | See *Methods* (Search strategy and study selection) and *Supplement data*. |
| Selection process | 8 | Specify the methods used to decide whether a study met the inclusion criteria of the review, including how many reviewers screened each record and each report retrieved, whether they worked independently, and if applicable, details of automation tools used in the process. | See *Methods* (Search strategy and study selection) and *Supplement data* |
| Data collection process | 9 | Specify the methods used to collect data from reports, including how many reviewers collected data from each report, whether they worked independently, any processes for obtaining or confirming data from study investigators, and if applicable, details of automation tools used in the process. | See *Methods* (Search strategy and study selection, Data collection). |
| Data items | 10a | List and define all outcomes for which data were sought. Specify whether all results that were compatible with each outcome domain in each study were sought (e.g. for all measures, time points, analyses), and if not, the methods used to decide which results to collect. | See *Methods* (Search strategy and study selection, Data collection, Statistics). |
|  | 10b | List and define all other variables for which data were sought (e.g. participant and intervention characteristics, funding sources). Describe any assumptions made about any missing or unclear information. | See *Methods* (Search strategy and study selection, Data collection, Statistics). |
| Study risk of bias assessment | 11 | Specify the methods used to assess risk of bias in the included studies, including details of the tool(s) used, how many reviewers assessed each study and whether they worked independently, and if applicable, details of automation tools used in the process. | See *Methods* (Quality assessment). |
| Effect measures | 12 | Specify for each outcome the effect measure(s) (e.g. risk ratio, mean difference) used in the synthesis or presentation of results. | See *Methods* (Search strategy and study selection, Data collection, Statistics). |
| Synthesis methods | 13a | Describe the processes used to decide which studies were eligible for each synthesis (e.g. tabulating the study intervention characteristics and comparing against the planned groups for each synthesis (item #5)). | See *Methods* (Search strategy and study selection, Data collection, Statistics). |
|  | 13b | Describe any methods required to prepare the data for presentation or synthesis, such as handling of missing summary statistics, or data conversions. | See *Methods* (Search strategy and study selection, Data collection, Statistics). |
|  | 13c | Describe any methods used to tabulate or visually display results of individual studies and syntheses. | See *Methods* (Search strategy and study selection, Data collection, Statistics). |
|  | 13d | Describe any methods used to synthesize results and provide a rationale for the choice(s). If meta-analysis was performed, describe the model(s), method(s) to identify the presence and extent of statistical heterogeneity, and software package(s) used. | See *Methods* (Search strategy and study selection, Data collection, Statistics). |
|  | 13e | Describe any methods used to explore possible causes of heterogeneity among study results (e.g. subgroup analysis, meta-regression). | See *Methods* (Search strategy and study selection, Data collection, Statistics). |
|  | 13f | Describe any sensitivity analyses conducted to assess robustness of the synthesized results. | See *Methods* (Search strategy and study selection, Data collection, Statistics). |
| Reporting bias assessment | 14 | Describe any methods used to assess risk of bias due to missing results in a synthesis (arising from reporting biases). | See *Methods* (Search strategy and study selection, Data collection, Statistics, Quality assessment). |
| Certainty assessment | 15 | Describe any methods used to assess certainty (or confidence) in the body of evidence for an outcome. | See *Methods* (Search strategy and study selection, Data collection, Statistics, Quality assessment). |
| **RESULTS** | | |  |
| Study selection | 16a | Describe the results of the search and selection process, from the number of records identified in the search to the number of studies included in the review, ideally using a flow diagram. | See *Results* (Characteristics of the included studies). |
|  | 16b | Cite studies that might appear to meet the inclusion criteria, but which were excluded, and explain why they were excluded. | See *Results* (Characteristics of the included studies). |
| Study characteristics | 17 | Cite each included study and present its characteristics. | See *Results* (Characteristics of the included studies, Summary of evidence for *in vitro* studies, Summary of evidence for *ex-vivo* studies, Summary of evidence for *in vivo* studies, Neuroprotective effect of Ar administration, Other organ protective effects of Ar administration, Safety of Ar administration, Meta-analysis on neuroprotective effect of Ar administration, Summary of evidence for clinical studies) and *Supplement data*. |
| Risk of bias in studies | 18 | Present assessments of risk of bias for each included study. | See *Results* (Meta-analysis on neuroprotective effect of Ar administration) and *Supplement data*. |
| Results of individual studies | 19 | For all outcomes, present, for each study: (a) summary statistics for each group (where appropriate) and (b) an effect estimate and its precision (e.g. confidence/credible interval), ideally using structured tables or plots. | See *Results* (Characteristics of the included studies, Summary of evidence for *in vitro* studies, Summary of evidence for *ex-vivo* studies, Summary of evidence for *in vivo* studies, Neuroprotective effect of Ar administration, Other organ protective effects of Ar administration, Safety of Ar administration, Meta-analysis on neuroprotective effect of Ar administration, Summary of evidence for clinical studies) and *Supplement data*. |
| Results of syntheses | 20a | For each synthesis, briefly summarise the characteristics and risk of bias among contributing studies. | See *Results* (Characteristics of the included studies, Summary of evidence for *in vitro* studies, Summary of evidence for *ex-vivo* studies, Summary of evidence for *in vivo* studies, Neuroprotective effect of Ar administration, Other organ protective effects of Ar administration, Safety of Ar administration, Meta-analysis on neuroprotective effect of Ar administration, Summary of evidence for clinical studies) and *Supplement data*. |
|  | 20b | Present results of all statistical syntheses conducted. If meta-analysis was done, present for each the summary estimate and its precision (e.g. confidence/credible interval) and measures of statistical heterogeneity. If comparing groups, describe the direction of the effect. | See *Results* (Characteristics of the included studies, Summary of evidence for *in vitro* studies, Summary of evidence for *ex-vivo* studies, Summary of evidence for *in vivo* studies, Neuroprotective effect of Ar administration, Other organ protective effects of Ar administration, Safety of Ar administration, Meta-analysis on neuroprotective effect of Ar administration, Summary of evidence for clinical studies) and *Supplement data*. |
|  | 20c | Present results of all investigations of possible causes of heterogeneity among study results. | See *Results* (Characteristics of the included studies, Summary of evidence for *in vitro* studies, Summary of evidence for *ex-vivo* studies, Summary of evidence for *in vivo* studies, Neuroprotective effect of Ar administration, Other organ protective effects of Ar administration, Safety of Ar administration, Meta-analysis on neuroprotective effect of Ar administration, Summary of evidence for clinical studies) and *Supplement data*. |
|  | 20d | Present results of all sensitivity analyses conducted to assess the robustness of the synthesized results. | See *Results* (Characteristics of the included studies, Summary of evidence for *in vitro* studies, Summary of evidence for *ex-vivo* studies, Summary of evidence for *in vivo* studies, Neuroprotective effect of Ar administration, Other organ protective effects of Ar administration, Safety of Ar administration, Meta-analysis on neuroprotective effect of Ar administration, Summary of evidence for clinical studies) and *Supplement data*. |
| Reporting biases | 21 | Present assessments of risk of bias due to missing results (arising from reporting biases) for each synthesis assessed. | See *Results* (Characteristics of the included studies, Summary of evidence for *in vitro* studies, Summary of evidence for *ex-vivo* studies, Summary of evidence for *in vivo* studies, Neuroprotective effect of Ar administration, Other organ protective effects of Ar administration, Safety of Ar administration, Meta-analysis on neuroprotective effect of Ar administration, Summary of evidence for clinical studies) and *Supplement data*. |
| Certainty of evidence | 22 | Present assessments of certainty (or confidence) in the body of evidence for each outcome assessed. | See *Results* (Characteristics of the included studies, Summary of evidence for *in vitro* studies, Summary of evidence for *ex-vivo* studies, Summary of evidence for *in vivo* studies, Neuroprotective effect of Ar administration, Other organ protective effects of Ar administration, Safety of Ar administration, Meta-analysis on neuroprotective effect of Ar administration, Summary of evidence for clinical studies) and *Supplement data*. |
| **DISCUSSION** | | |  |
| Discussion | 23a | Provide a general interpretation of the results in the context of other evidence. | See *Discussion*. |
|  | 23b | Discuss any limitations of the evidence included in the review. | See *Discussion*. |
|  | 23c | Discuss any limitations of the review processes used. | See *Discussion*. |
|  | 23d | Discuss implications of the results for practice, policy, and future research. | See *Discussion*. |
| **OTHER INFORMATION** | | |  |
| Registration and protocol | 24a | Provide registration information for the review, including register name and registration number, or state that the review was not registered. | See *Methods* (Search strategy and study selection). |
|  | 24b | Indicate where the review protocol can be accessed, or state that a protocol was not prepared. | See *Methods* (Search strategy and study selection). |
|  | 24c | Describe and explain any amendments to information provided at registration or in the protocol. | See *Methods* (Search strategy and study selection). |
| Support | 25 | Describe sources of financial or non-financial support for the review, and the role of the funders or sponsors in the review. | See *Methods* (Role of Funders source) and *Acknowledgments*. |
| Competing interests | 26 | Declare any competing interests of review authors. | See *Declaration of interests*. |
| Availability of data, code and other materials | 27 | Report which of the following are publicly available and where they can be found: template data collection forms; data extracted from included studies; data used for all analyses; analytic code; any other materials used in the review. | See *Data sharing statement.* |

**Table S2. PRISMA 2020 for abstract checklist**

| **Section and Topic** | **Item #** | **Checklist item** | **Reported (Yes/No)** |
| --- | --- | --- | --- |
| **TITLE** | | |  |
| Title | 1 | Identify the report as a systematic review. | Yes |
| **BACKGROUND** | | |  |
| Objectives | 2 | Provide an explicit statement of the main objective(s) or question(s) the review addresses. | Yes |
| **METHODS** | | |  |
| Eligibility criteria | 3 | Specify the inclusion and exclusion criteria for the review. | Yes |
| Information sources | 4 | Specify the information sources (e.g. databases, registers) used to identify studies and the date when each was last searched. | Yes |
| Risk of bias | 5 | Specify the methods used to assess risk of bias in the included studies. | Yes |
| Synthesis of results | 6 | Specify the methods used to present and synthesise results. | Yes |
| **RESULTS** | | |  |
| Included studies | 7 | Give the total number of included studies and participants and summarise relevant characteristics of studies. | Yes |
| Synthesis of results | 8 | Present results for main outcomes, preferably indicating the number of included studies and participants for each. If meta-analysis was done, report the summary estimate and confidence/credible interval. If comparing groups, indicate the direction of the effect (i.e. which group is favoured). | Yes |
| **DISCUSSION** | | |  |
| Limitations of evidence | 9 | Provide a brief summary of the limitations of the evidence included in the review (e.g. study risk of bias, inconsistency and imprecision). | Yes |
| Interpretation | 10 | Provide a general interpretation of the results and important implications. | Yes |
| **OTHER** | | |  |
| Funding | 11 | Specify the primary source of funding for the review. | Yes |
| Registration | 12 | Provide the register name and registration number. | Yes |

**Search strategy for systematic review**

The search was performed on August 4^th^, 2023, and subsequently updated on March 15^th^, 2024. The data delineated in ***Table S3-S5*** corresponds to the most recent iteration of the search. The search strategy involved the utilization of two distinct algorithms. The first algorithm (detailed in ***Table S3***) was applied to identify clinical and pre-clinical (*in vivo* and *ex vivo*) studies investigating the potential protective effects of Argon (Ar) under specific clinical conditions. Simultaneously, the second algorithm (***Table S4***) was used to detect relevant *in vitro* studies examining the potential effects of Ar on cellular mechanisms. Subsequently, a combination of these two algorithms was executed to comprehensively identify all pertinent scientific literature of interest (***Table S5***). Finally, we also performed a snowball search to identify additional studies by searching the reference lists of publications eligible for full-text review.

**Table S3. Search algorithm 1 - Search for clinical and pre-clinical (*in vivo* and *ex-vivo*) studies**

| **#** | **Search term** | **Counts** |
| --- | --- | --- |
| 1 | "argon"[MeSH Terms] OR "argon"[All Fields] | 19,736 |
| 2 | "protect*"[All Fields] OR "neuroprotect*"[All Fields] OR "neuro protect*"[All Fields] OR ("neuro"[All Fields] AND "protect*"[All Fields]) OR "cardioprotect*"[All Fields] OR "cardio protect*"[All Fields] OR ("cardio"[All Fields] AND "protect*"[All Fields]) OR "organprotect*"[All Fields] OR "organ protect*"[All Fields] OR ("organ"[All Fields] AND "protect*"[All Fields]) | 1,285,822 |
| 3 | #1 AND #2 | 539 |
| 4 | (("ischemi*"[All Fields] AND "encephalopath*"[All Fields]) OR ("ischaemi*"[All Fields] AND "encephalopath*"[All Fields]) OR ("encephalopath*"[All Fields] AND "hypoxia*"[All Fields]) OR ("brain*"[All Fields] AND "hypoxia*"[All Fields]) OR ("ischemi*"[All Fields] AND "brain*"[All Fields]) OR ("ischaemi*"[All Fields] AND "brain*"[All Fields]) OR "hypoxia ischemia, brain"[MeSH Terms]) AND "perinatal*"[All Fields] | 4,637 |
| 5 | #3 AND #4 | 9 |
| 6 | ("heart"[All Fields] AND "arrest"[All Fields]) OR ("cardiac"[All Fields] AND "arrest"[All Fields]) OR ("cardiopulmonary"[All Fields] AND "arrest"[All Fields]) OR "heart arrest"[MeSH Terms] | 93,781 |
| 7 | #3 AND #6 | 18 |
| 8 | "ischemic stroke"[MeSH Terms] OR ("ischemi*"[All Fields] AND "stroke*"[All Fields]) OR ("ischaemi*"[All Fields] AND "stroke*"[All Fields]) OR ("ischemi*"[All Fields] AND "brain*"[All Fields]) OR ("ischaemi*"[All Fields] AND "brain*"[All Fields]) | 198,108 |
| 9 | #3 AND #8 | 61 |
| 10 | "subarachnoid hemorrhage"[MeSH Terms] OR ("hemorrhage*"[All Fields] AND "subarachnoid*"[All Fields]) OR ("haemorrhage*"[All Fields] AND "subarachnoid*"[All Fields]) | 38,176 |
| 11 | #3 AND #10 | 8 |
| 12 | "TBIs"[All Fields] OR "TBI"[All Fields] OR ("trauma*"[All Fields] AND "brain*"[All Fields]) OR "brain injuries, traumatic"[MeSH Terms] | 109,117 |
| 13 | #3 AND #12 | 22 |
| 14 | "multiple organ failure"[MeSH Terms] OR "multiple organ failure"[All Fields] OR ("multiple"[All Fields] AND "organ*"[All Fields] AND "failure"[All Fields]) OR "multiple organ dysfunction"[All Fields] OR ("multiple"[All Fields] AND "organ*"[All Fields] AND "dysfunction*"[All Fields]) OR "MODS"[All Fields] | 37,142 |
| 15 | #3 AND #14 | 2 |
| 16 | "retina*"[All Fields] AND ("reperfusion injury"[MeSH Terms] OR ("reperfusion*"[All Fields] AND "injury*"[All Fields]) OR ("ischemi*"[All Fields] AND "ischaemi*"[All Fields])) | 1,525 |
| 17 | #3 AND #16 | 7 |
| 18 | #5 OR #7 OR #9 OR #11 OR #13 OR #15 OR #17 | 80 |

**Table S4. Search algorithm 2 - Search for *in vitro* studies**

| **#** | **Search term** | **Counts** |
| --- | --- | --- |
| 1 | "argon"[MeSH Terms] OR "argon"[All Fields] | 19,736 |
| 2 | "vitro"[All Fields] AND (“oxygen glucose deprivation”[All Fields] OR “OGD”[All Fields]) | 3,732 |
| 3 | #1 AND #2 | 13 |
| 4 | "vitro"[All Fields] AND (“injury”[All Fields]) | 65,161 |
| 5 | #1 AND #4 | 59 |
| 6 | "ex-vivo"[All Fields] OR "transplantation"[All Fields] OR "autotransplantation"[All Fields] OR "preconditioning"[All Fields] OR "postconditioning"[All Fields] | 983,311 |
| 7 | #1 AND #6 | 276 |
| 8 | #3 OR #5 OR #7 | 330 |

**Table S5. Combination of two search algorithms**

| **#** | **Search term** | **Counts** |
| --- | --- | --- |
| 1 | #18 (see ***Table S3***) OR #8 (see ***Table S4***) | 380 |

**Details for outcomes included in the meta-analysis of neuroprotective effect of Ar in *in vivo* studies**

The neuroprotective effect of Ar was assessed by examining multiple endpoints that provide a comprehensive description across various domains. These encompass functional aspects, including neurological, cognitive and locomotor measures, physiological parameters such as haemodynamic, metabolic, and respiratory exchange metrics, as well as histological markers, specifically focusing on neurodegeneration and inflammation (***Table S6***).

**Table S6. Endpoints used for the assessment of Argon neuroprotective effect**

| **Domain** | **Outcome** | **Endpoint (measure)** | **Time point** |
| --- | --- | --- | --- |
| **Functional** | Neurological | Neurological Deficit Score | After 24 hours and < 4 days from the end of treatment administration |
|  | Cognitive and locomotor | - Barnes maze test - Morris water-maze test - Taper removal test - Rotarod test - Vertical pole test - Beam walking test - Open Field test | After 24 hours and < 4 days from the end of treatment administration |
| **Physiological** | Haemodynamic | - Mean Arterial Pressure (MAP) - Heart Rate (HR) - Cardiac output (CO) | At the end of treatment administration or within 24 hours from the end of treatment administration |
|  | Metabolic | - Base Excess (BE) - Lactate - pH - Glucose | At the end of treatment administration or within 24 hours from the end of treatment administration |
|  | Respiratory exchange | - Arterial partial pressure of oxygen (PaO_2_) - Arterial partial pressure of carbon dioxide (PaCO_2_) | At the end of treatment administration or within 24 hours from the end of treatment administration |
| **Histological** | Neurodegeneration | Count of cells on mm^2^ evaluated for: hippocampus (right, left, CA1, CA2/3, CA3, CA 3/4), cortex, penumbra, ischemic core, and subcortex.  Staining methods include CV, NeuN, HE, TUNEL, and FJB. | End of experiment |
|  | Inflammation | Count of cells on mm^2^ evaluated for: hippocampus (right, left, CA1, CA3, CA2/3, CA3, CA 3/4), cortex, penumbra, ischemic core, and subcortex.  Staining methods include: Iba1 and F4/80 to detect microglia and microglia extension respectively, GFAP to detect Astrocytes. | End of experiment |

*Abbreviations. CV, Cresyl Violet; FJB, Fluoro Jade B; HE, Hematoxylin Eosin; GFAP, glial fibrillar acid protein; NeuN, Neuronal Nuclei Antigen; TUNEL, terminal deoxynucleotidyl transferase dUTP nick-end labeling.*

**Characteristics of studies included in the systematic review and in the meta-analysis**

The systematic review included a total of 61 studies, comprising one clinical study and 60 pre-clinical studies. The main characteristics of the 60 pre-clinical studies are detailed in ***Table S7*** (*in vitro* studies) and ***Table S8*** (*in vivo* and *ex vivo* studies). Six studies are listed in both ***Table S7*** and ***Table S8***, because they included both *in vivo* and *in vitro* experiments.

The *in vivo* studies included in the meta-analysis are reported in ***Table S9***, together with a summary of their risk of bias. The *in vitro* studies included in the meta-analysis are reported in ***Table S10***, together with a summary of their risk of bias.

**Table S7. Characteristics of *in vitro* studies included in the systematic review**

| **Article**  **(year)** | **Cell**  **Tissue** | **Model** | **EXP group** | **CTR group** | **Study aim** | | | | | **Study conclusion on Ar effect** | | | | |
| --- | --- | --- | --- | --- | --- | --- | --- | --- | --- | --- | --- | --- | --- | --- |
|  |  |  |  |  | **Neuro**  **protection** | **Organ**  **protection** | **Cardio**  **protection** | **Molecular**  **mechanisms** | **Safety** | **Neuro**  **protection** | **Organ**  **protection** | **Cardio**  **protection** | **Molecular**  **mechanisms** | **Safety** |
| Yarin  et al., 2005^1^ | Rat organ of Corti cultures | 1. Hypoxia  2. Drug induced cell injury - Cisplatin  3. Drug induced cell injury - Gentamycin | 1. Ar/CO_2_  95/5, for 30h  2. Ar/O_2_/CO_2_  74/21/5, for 48h  3. Ar/O_2_/CO_2_  74/21/5, for 48h | 1.Normoxia for 30h  2. N_2_/O_2_/CO_2_ 74/21/5,  for 48h  3. Ar/O_2_/CO_2_ 74/21/5,  for 48h |  | **✔** |  |  |  |  | **+** |  |  |  |
| Jawad  et al., 2009^2^ | Foetal mice Neuronal cells | Cell injury - OGD + Hypoxia | Ar/N_2_/CO_2_  75/20/5 | N_2_/CO_2_  95/5 | **✔** |  |  | ✔ |  | **+** |  |  | **+** |  |
| Loetscher  et al., 2009^3^ | Hippocampal Slices mice | 1. Cell injury - OGD  2. TBI above Hp | Ar/O_2_/N_2_  (25, 50, 74)/21/balance with N_2_ | N_2_/O_2_/CO_2_  74/21/5 | **✔** |  |  |  |  | **+** |  |  |  |  |
| Rizvi  et al., 2010^4^ | Human tubular kidney cells (HK2) | Cell injury - OGD | Ar/O_2_/CO_2_ 75/20/5, for 3h | N_2_/CO_2_  95/5 |  | **✔** |  | ✔ |  |  | **-** |  | **+** |  |
| David  et al., 2012^5^ | Male rats brain coronal slices | Cell injury - OGD | Ar/O_2_/N_2_  (25, 30, 37.5, 50, 75)/25/ balance with N_2_ | N_2_/O_2_  75/25 | ✔ |  |  | **✔** |  | **=** |  |  | **+** |  |
| Fahlenkamp  et al., 2012^6^ | Primary neuronal and astroglial cell cultures and the microglial cell line (BV-2) mice | Selective influence of Ar on ERK 1/2 | Ar/N_2_/O_2_/CO_2_ 50/24/21/5 for 15 or 30 or 60 or 120 min | N_2_/O_2_/CO_2_  74/21/5 |  |  |  | **✔** |  |  |  |  | **+** |  |
| Harris  et al., 2013^7^ | Mice Hippocampal brain slices | Focal mechanical trauma | Ar 50, 30 min to 24h | N_2_/O_2_/CO_2_  75/20/5 | **✔** |  |  | ✔ |  | **+** |  |  | **+** |  |
| David  et al., 2013^8^ | Male Sprague–Dawley mature rats whole-blood samples | 1. Catalytic efficiency: tPA 2. Thrombolytic efficiency: whole blood (rats) | Ar/O_2_/N_2_ (25, 30, 37.5, 50, 75)/25/ balance with N2 | N_2_/O_2_  75/25 |  |  |  | **✔** |  |  |  |  | **+** |  |
| Spaggiari  et al., 2013^9^ | Human osteosarcoma cells (U2OS) | Drug induced cell injury - STS, MTX, Rotenone, Antimycin A, menadione | Ar/O_2_/CO_2_ 75/20/5 | No  intervention |  |  |  | **✔** |  |  |  |  | **+** |  |
| Ulbrich  et al., 2015^10^ | Human neuroblastoma cells (SH-SY5Y) | Drug induced cell injury - Rotenone | Ar/O_2_/CO_2_/N_2_  (25, 50, 75)/21/5/balance with N_2_, for 2 or 4 hours | N_2_/O_2_/CO_2_  (25, 50, 75)/21/5,  for 2 or 4 hours | **✔** |  |  | **✔** |  |  |  |  | **+** |  |
| Ulbrich  et al., 2016^11^ | Human neuroblastoma cells (SH-SY5Y) | Drug induced cell injury - Rotenone | After 4h of rotenone-treatment, exposed to Ar 75%, for 2h | After 4h of rotenone-treatment, exposed to room air, for 2h |  |  |  | **✔** |  |  |  |  | **+** |  |
| Zhao  et al., 2016^12^ | Rat cortical neuronal cells | Cell injury - OGD | Ar/CO_2_/O_2_  70/5/  balance with O_2_, at 33°C for 2h | N_2_/CO_2_/O_2_  70/5/balance with O2, at 33°C for 2h | **✔** |  |  | ✔ |  | **+** |  |  | **+** |  |
| Zhao  et al., 2016^13^ | Rat cortical neuronal cells | Cell injury - OGD | Ar/CO_2_/N_2_  70/5/  balance with N_2_, for 2h | O_2_/CO_2_/N_2_  70/5/balance with N_2_, for 2h | **✔** |  |  | ✔ |  | **+** |  |  | **+** |  |
| Hafner  et al., 2016^14^ | Human airway epithelial cells (A549) | Cytotoxic concentration of H_2_O_2_ | H_2_O_2_ exposure to Ar/O_2_/CO_2_/N_2_  50 or 30  /21/5/balance with N_2_,  for 30 or 45 or 180 min | No  intervention  or  H_2_O_2_ exposure after standard air for 30, 45 or 180 min |  | **✔** |  | ✔ |  |  | **+** |  | **+** |  |
| Mayer  et al., 2016^15^ | Primary isolated cardiomyocytes from neonatal rats | Hypoxia | Ar/N_2_/O_2_/CO_2_  50/24/21/5  for 1 h and then exposed to hypoxia for 5h | No  intervention |  |  | **✔** | ✔ |  |  |  | **+** | **+** |  |
| Lemoine  et al., 2017^16^ | 1. Cardiomyocytes  2. Human right atrial appendage | Hypoxia/Reoxygenation | Ar/N_2_  70/30  administered during the last 2min of hypoxia followed by Ar/O_2_ 70/30 during the first 3min of reoxygenation. | Hypoxia/Reoxygenation protocol alone |  |  | **✔** | ✔ |  |  |  | **+** | **+** |  |
| Grüßer  et al., 2017^17^ | Mice hippocampal brain slices | Traumatic brain injury | Ar 50,  2h | No  intervention | **✔** |  |  |  |  | **-** |  |  |  |  |
| Qi  et al., 2018^18^ | Human cardiac myocyte-like progenitor cells (HCMs) isolated from explanted human hearts derived from heart transplantation surgery | Cell injury - OGD | Ar/O_2_/CO_2_/N_2_  50 or 30  /21/5/balance with N_2_, for 1h30min | O_2_/CO_2_/N_2_  21/5/balance with N_2_, for 1h30min |  |  | **✔** | ✔ |  |  |  | **+** | **+** |  |
| Koziakova eta al., 2019^19^ | Organotypic hippocampal brain slices from male and female mice | Hypoxia-Ischemia - OGD | Ar 50 | N_2_/O_2_/CO_2_ 75/20/5 | **✔** |  |  | ✔ |  | + |  |  | **+** |  |
| Qi  et al., 2021^20^ | Cardiomyocytes H2C9 | Cell injury – OGD | 1.(Ar pre-treatment group) cultured with Ar 50,  1h, incubated with N_2_/O_2_/CO_2_ 74/21/5  2. (Ar + short term hypoxia group) cultured with Ar50,  1h, incubated with N_2_/O_2_/CO_2_ 94/1/5 for 5h  3. (Ar + long term hypoxia group) cultured with Ar50,  1h, incubated with N_2_/O_2_/CO_2_ 94/1/5 for 10h | 1.(Normal group)  N_2_/O_2_/CO_2_  74/21/5  2. (Short term hypoxia group)  N_2_/O_2_/CO_2_  94/1/5  for 5h  3. (Long term hypoxia group)  N_2_/O_2_/CO_2_  94/1/5 for 10h |  |  | **✔** | ✔ |  |  |  | **+** | **+** |  |
| Goebel  et al., 2021^21^ | Human Neuroblastoma Cells (SH-SY5Y) | Drug induced cell injury - Rotenone | Rotenone  for 4h and then immediately exposed for 2h Ar 75% | Rotenone for 4h and then immediately exposed for 2h room air | **✔** |  |  | ✔ | ✔ | **+** |  |  | **+** | **+** |
| Scheid  et al., 2023^22^ | Human Neuroblastoma Cells (SH-SY5Y) | Drug induced cell injury - Rotenone | Ar/CO_2_/N_2_  (74, 50, 25)/5/ balance with N_2_, for 2h or 4h | Rotenone induced cell injury only |  |  |  | **✔** |  |  |  |  | **+** |  |

*Abbreviations. Ar, Argon; CO2, Carbon dioxide; CTR, control; EXP, experimental; Hp, Hippocampus; N_2_, nitrogen; O_2_, oxygen; OGD, Oxygen Glucose Deprivation.*

*Symbols. ✔, secondary aims;* ***✔****, primary study aim; +, positive/supporting; =, neutral; -, negative/opposite.*

**Table S8. Characteristics of *in vivo* and *ex vivo* studies included in the systematic review**

| **Article**  **(year)** | **Study Design** | **Disease**  **or**  **Organ** | **Model** | **Animal model** | **N**  **per**  **group** | **EXP Group** | **CTR Group** | **Study aim** | | | | | **Study conclusion on Ar effect** | | | | |
| --- | --- | --- | --- | --- | --- | --- | --- | --- | --- | --- | --- | --- | --- | --- | --- | --- | --- |
|  |  |  |  |  |  |  |  | **Neuro**  **protection** | **Organ**  **protection** | **Cardio**  **protection** | **Molecular**  **mechanisms** | **Safety** | **Neuro**  **protection** | **Organ**  **protection** | **Cardio**  **protection** | **Molecular**  **mechanisms** | **Safety** |
| Pagel  et al., 2007^23^ | RCT | Myocardial Infarction | LAD  occlusion | Male New Zealand white rabbits  (2.5-3.0 kg) | 8 | Ar/O_2_ 70/30,  3 cycles of 5 mins each one (15 mins),  5 min pre-I | 0.9% saline,  3 cycles of 5 mins each one (15 mins),  5 min pre-I |  |  | **✔** | ✔ |  |  |  | **+** | **+** |  |
| Ryang  et al., 2011^24^ | CT | Ischemic Stroke | tMCAO | Male adult  SD rats  (250-295 g) | 11 | Ar/O_2_ 50/50,  1h,  1h post-I | N_2_/O_2_ 50/50,  1h,  1h post-I | **✔** |  |  |  | ✔ | **+** |  |  |  | **+** |
| David  et al., 2012^5^ | RCT | Ischemic Stroke | MCAO | Male adult  SD rats  (250-280 g) | 7-11 | Ar/O_2_/N_2_ (25- 37.5 -50 - 75) /25%/balance with N_2_,  3h,  1h post-I | N_2_/O_2_ 75/25,  3h,  1h post-I | **✔** |  |  |  | ✔ | **=** |  |  |  | **+** |
| Zhuang  et al., 2012^25^ | RCT | Perinatal HIE | Carotid artery ligation | Postnatal (7 days)  SD rats | 6 | Ar/O_2_ 70/30,  1:30h,  2h post-I | N_2_/O_2_ 70/30,  3h,  1h post-I | **✔** |  |  | ✔ |  | **+** |  |  | **+** |  |
| Brucken  et al., 2013^26^ | RCT | Cardiac arrest | VF by transoesophageal electrical stimulation | Male  SD rats  (400-500 g) | 7 | Ar/O_2_ 70/30,  1h,  1h post-I | N_2_/O_2_ 70/30,  1h,  1h post-I | **✔** |  |  |  | ✔ | **+** |  |  |  | **+** |
| Brucken  et al., 2014^27^ | RCT | Cardiac arrest | VF by transoesophageal electrical stimulation | Male  SD rats  (400-500 g) | 18 | Ar/O_2_ 70/30  Ar/O_2_/N_2_ 40/30/30,  1h,  1h post-I | N_2_/O_2_ 70/30,  1h,  1h post-I | **✔** |  |  |  | ✔ | **+** |  |  |  | **+** |
| Fahlenkamp  et al., 2014^28^ | RCT | Ischemic Stroke | tMCAO | Male adult  SD rats  (250-295 g) | 15 | Ar/O_2_ 50/50,  1h,  1h post-I | N_2_/O_2_ 50/50,  1h,  1h post-I | **✔** |  |  | ✔ | ✔ | **=** |  |  | **+** | **+** |
| Ulbrich  et al., 2014^29^ | RCT | Retinal IRI | Anterior chamber hypertension | Adult male and female  SD rats  (280-350 g) | 24 | Ar/O_2_ (25-50-75%)/O_2_ (21%)/balance with N_2_,  1h,  immediately or 1:30h or 3h post-I | Room air,  1h,  immediately or 1:30h or 3h post-I | **✔** |  |  | ✔ |  | **+** |  |  | **+** |  |
| Ristagno  et al., 2014^30^ | RCT | Cardiac arrest | LAD occlusion  + VF | Male  domestic pigs  (38-39 kg) | 6 | Ar/O2 70/30,  4h,  immediately post-I | N_2_/O_2_ 70/30,  4h,  immediately post-I | **✔** |  |  |  | ✔ | **+** |  |  |  | **+** |
| Alderliesten  et al., 2014^31^ | RCT | Perinatal HIE | Hypoxia | Newborn  piglets  (1.4-3.0 kg) | 2 | Ar (30-50-80) balance with O_2_,  3 cycles of 60 mins each (180 mins),  30min post-I | No treatment |  |  |  |  | **✔** |  |  |  |  | **+** |
| Brucken  et al., 2015^32^ | RCT | Cardiac arrest | VF by transoesophageal electrical stimulation | Male  SD rats  (400-500 g) | 16 | Ar/O_2_ 70/30,  1h,  1h or 3h post-I | N2/O2 70/30,  1h,  1h or 3h post-I | **✔** |  |  |  | ✔ | **+** |  |  |  | **+** |
| Ulbrich  et al., 2015^33^ | RCT | Retinal IRI | Anterior chamber hypertension | Adult male and female SD rats (280-350 g) | 24 | Ar/O_2_/N_2_ 75/21/4,  1h,  immediately or 1:30h or 3h post-I | Room air,  1h,  immediately or 1:30h or 3h post-I | ✔ |  |  | **✔** |  | **+** |  |  | **+** |  |
| Ulbrich  et al., 2016^11^ | RCT | Retinal IRI | Anterior chamber hypertension | Adult male and female  SD rats  (280-350 g) | 8 | Ar/O_2_/N_2_ 75/21/4,  1h,  1h post-I | Room air,  1h,  1h post-I | **✔** |  |  | ✔ |  | **+** |  |  | **+** |  |
| Zhao  et al., 2016^13^ | RCT | Perinatal HIE | Unilateral common carotid artery ligation | 7-day-old  rats | 8 | Ar/O_2_ 70/30,  2h,  immediately post-I | N_2_/O_2_ 70/30, 2h, immediately post-I | **✔** |  |  | ✔ |  | **+** |  |  | **+** |  |
| Zhao  et al., 2016^12^ | RCT | Perinatal HIE | Unilateral common carotid artery ligation + Hypoxia | 7-day-old  SD rat | 12 | Ar/O_2_ 70/30,  2h,  2h post-I + MTH | N_2_/O_2_ 70/30,  2h,  2h post-I + MTH | **✔** |  |  | ✔ |  | **+** |  |  | **+** |  |
| Broad  et al., 2016^34^ | RCT | Perinatal HIE | Bilateral common carotid artery occlusion | Newborn (<40 hs) male  large white piglets | 8 | Ar (45-48-50) balance with O_2_ and N2,  24h,  2h post-I + MTH | Cooling (MTH),  24h,  2h post-I | **✔** |  |  |  | ✔ | **+** |  |  |  | **+** |
| Hollig  et al., 2016^35^ | RCT | SAH | Endovascular perforation technique | Male SD rats  (300-400 g) | 27 | Ar/O_2_ 50/50,  1h,  1h post-I | N_2_/O_2_ 50/50,  1h,  1h post-I | **✔** |  |  | ✔ |  | **+** |  |  | **+** |  |
| Zuercher  et al., 2016^36^ | RCT | Cardiac arrest | KCl + Esmolol | Adult (9-10 weeks) male  Wistar rats | 10 | Ar/O_2_ 50/50,  24h,  15min post-I | N_2_/O_2_ 50/50,  24h,  15min post-I | **✔** |  |  |  | ✔ | **-** |  |  |  | **+** |
| Ulmer  et al., 2017^37^ | RCT | Liver regeneration | Partial Hepatectomy | Adults (12-14 weeks) male  SD rats | 8 | Ar/O_2_ 50/50,  1h,  1h pre-I | N_2_/O_2_ 50/50,  1h,  1h pre-I |  | **✔** |  | ✔ |  |  | **-** |  | **=** |  |
| Lemoine  et al., 2017^16^ | RCT | Myocardial Infarction | LAD  ligation | Male  Wistar rats (240-380 g) | 8 | Ar/O_2_ 80/20,  20min,  5min pre-I | N_2_/O_2_ 80/20,  20min,  5min pre-I |  |  | **✔** |  |  |  |  | **+** |  |  |
| Brucken  et al., 2017^38^ | RCT | Cardiac arrest | VF by transoesophageal electrical stimulation | Male SD rats  (400-500 g) | 7 | Ar/O_2_ 70/30,  1h,  1h post-I + MTH | N_2_/O_2_ 70/30,  1h,  1h post-I + MTH | **✔** |  |  |  | ✔ | **+** |  |  |  | **+** |
| Savary  et al., 2018^39^ | RCT | MODS | Supra-coeliac aortic cross-clamping | Male  New Zealand rabbits  (2.5-3.5 kg) | 24 | Ar/O_2_ 70/30,  1:30h or 2h or 5:30h,  30 min pre-I or immediately post-I | N_2_/O_2_ 70/30,  1:30h or 2h or 5:30h,  30 min pre-I or immediately post-I |  | **✔** |  |  | ✔ |  | **+** |  |  | **+** |
| Ma  et al., 2019^40^ | RCT | Ischemic Stroke | pMCAO or tMCAO  focal cerebral ischemia via MCAO | Adult male  Wistar rats | 16 | Ar/O_2_ 70/30,  24h,  immediately post-I | N_2_/O_2_ 70/30,  24h,  immediately post-I | **✔** |  |  |  | ✔ | **+** |  |  |  | **+** |
| Liu  et al., 2019^41^ | RCT | Ischemic Stroke | tMCAO | Adult male  Wistar rats | 8 | Ar/O_2_ 50/50,  1h,  1h post-I | N_2_/O_2_ 50/50,  1h,  1h post-I | **✔** |  |  | ✔ |  | **+** |  |  | **+** |  |
| Kremer  et al., 2020^42^ | RCT | SAH | Modified endovascular perforation model | Adult (9 weeks) male  SD rats  (300-400 g) | 17 | Ar/O_2_ 50/50,  1h,  1h post-I | N_2_/O_2_ 50/50,  1h,  1h post-I | **✔** |  |  |  | ✔ | **=** |  |  |  | **+** |
| Schmitz  et al., 2020^43^ | RCT | Ischemic Stroke | Hepatic IRI | Adult (12-14 weeks) male  SD rats | 24 | Ar/O_2_/Isofluorane 50/48/2,  1h,  1h pre-I | N_2_/O_2_/Isofluorane 50/48/2,  1h,  1h pre-I |  | **✔** |  |  | ✔ |  | **-** |  |  | **=** |
| Fumagalli  et al., 2020^44^ | RCT | Cardiac arrest | LAD Occlusion + VF | Male  Domestic pigs  (39 ± 2 kg) | 24 | Ar/O_2_ 70/30  Ar/O_2_/N_2_ 50/30/20,  4h,  immediately post-I | N_2_/O_2_ 70/30,  4h,  immediately post-I | **✔** |  | ✔ |  | ✔ | **+** |  | **+** |  | **+** |
| Goebel  et al., 2021^21^ | RCT | Retinal IRI | Anterior chamber hypertension | Adult male and female  SD rats  (280-350 g) | 6 | Ar/O_2_/N_2_ 75/21/4,  2h,  immediately post-I | Room air,  2h,  immediately post-I | **✔** |  |  | ✔ |  | **+** |  |  | **+** |  |
| Moro  et al., 2021^45^ | RCT | TBI | CCI | Adult male (9 weeks) C57BL/6J mice | 10 | Ar/O_2_ 70/30,  24h,  10min post-I | N_2_/O_2_ 70/30,  24h,  10min post-I | **✔** |  |  |  |  | **+** |  |  |  |  |
| Creed  et al., 2021^46^ | RCT | TBI | CHI | Adult male  C57/black mice | 55 | Ar/O_2_ 70/30  Ar/O_2_ 79/21,  24h,  30min post-I | N_2_/O_2_ 70/30 N2/O2 79/21,  24h,  30min post-I | **✔** |  |  |  |  | **-** |  |  |  |  |
| de Roux  et al., 2021^47^ | RCT | MODS | Supra-coeliac aortic cross-clamping | Male  New Zealand rabbits  (2.0-3.0 kg) | 7 | Ar/O_2_ 70/30,  330min,  30min pre-I | N_2_/O_2_ 70/30,  330min,  30min pre-I |  | **✔** |  |  | ✔ |  | **+** |  |  | **+** |
| Schneider  et al., 2022^48^ | RCT | TBI | CCI | Male  C57Bl/6 mice | 28 | Ar/O_2_ 75/25  Ar/O_2_ 50/50  Ar/N2/O_2_ 50/30/20  Ar/O_2_ 25/75,  4h,  30min pre-I | Room air,  15min or 24h,  30 min pre-I | **✔** |  |  |  | ✔ | **=** |  |  |  | **+** |
| He  et al., 2022^49^ | RCT | Ischemic Stroke | tMCAO | Male  ICR mice | NA | Ar/O_2_ 79/21  Ar/N_2_/O_2_ 39/40/21,  1h or 3h or 24h,  1h post-I | Sham,  1h or 3h or 24h,  1h post-I | **✔** |  |  |  |  | **+** |  |  |  | **+** |
| Antonova  et al., 2022^50^ | RCT | TBI | Open brain contusion injury | Male  Wistar Rats | 14 | Ar/O_2_ 70/30,  3h,  15 or 30min post-I | N_2_/O_2_ 70/30,  3h,  15 or 30min post-I | **✔** |  |  |  |  | **-** |  |  |  |  |
| Xue  et al., 2023^51^ | RCT | Ischemic Stroke | tMCAO | Male  ICR mice | NA | Ar/O_2_ 79/21,  3h,  1h post-I | N_2_/O_2_ 79/21,  3h,  1h post-I | **✔** |  |  |  |  | **+** |  |  |  |  |
| Liu  et al., 2022^52^ | RCT | Ischemic Stroke | tMCAO | Male  Wistar rats | 7 | Ar/O_2_ 50/50,  1h,  1h post-I | N_2_/O_2_ 50/50,  1h,  1h post-I | **✔** |  |  |  | ✔ | **+** |  |  |  | **+** |
| Silachev et al., 2023^53^ | RCT | Ischemic Stroke | Photochemically induced thrombosis of cerebral cortex vessels | Male  Wistar Rats | 10 (CTR)  8 (EXP) | Ar/O_2_ 70/30,  3 cycles of 2h each (6h),  15min post-I, 26h post-I, 72h post-I | N_2_/O_2_ 70/30,  3 cycles of 2h each (6h),  15min post-I, 26h post-I, 72h post-I | **✔** |  |  |  | ✔ | **+** |  |  |  | **+** |
| Irani  et al., 2012^54^ | RCT | Kidney | IRI + Renal transplantation | Male inbred  Wistar rats  (200-250 g) | 15 | Cold-storage solution saturated with  Ar 100%,  6h | Cold-storage solution saturated with Air,  6h |  | **✔** |  |  | ✔ |  | **+** |  |  | **+** |
| Faure  et al., 2016^55^ | RCT | Kidney | IRI + Renal transplantation | Female  pigs | 8 | Preservation solution saturated saturated with  Ar 100%,  30h | Preservation solution saturated saturated with Air,  30h |  | **✔** |  |  |  |  | **+** |  |  |  |
| Martens  et al., 2016^56^ | RCT | Lung | Warm  Ischemia  (2 hs) | Male  domestic male pigs  (36-42 kg) | 5 | EVLP:  Ar/O_2_ 70/30,  6h | EVLP:  N_2_/O_2_ 70/30, 6h |  | **✔** |  |  | ✔ |  | **-** |  |  | **+** |
| Martens  et al., 2017^57^ | RCT | Lung | Cold  Ischemia  (18 hs) | Male  domestic pigs | 6 | Pre/Per conditioning:  Ar/O_2_ 79/21,  24h Perfusion on EVLP;  Postconditioning:  Ar/O_2_ 88/12,  4h | Pre/Per conditioning: N_2_/O_2_ 79/21,  24h Perfusion on EVLP,  Postconditioning:  N_2_/O_2_ 88/12,  4h |  | **✔** |  |  | ✔ |  | **-** |  |  | **+** |
| Smith  et al., 2017^58^ | RCT | Kidney | IRI | Large white female  pigs  (50-60 kg) | 6 | EVNP:  Ar/O_2_/CO_2_ 70/25/5 | EVNP:  O_2_/CO_2_ 95/5 |  | **✔** |  |  | ✔ |  | **-** |  |  | **+** |
| De Deken  et al., 2018^59^ | RCT | Kidney | IRI | Female prepubescent (3–4 months)  pigs | 6 | Ar/O_2_ 70/30,  for 2h | N_2_/O_2_ 70/30,  for 2h |  | **✔** |  |  |  |  | **-** |  |  |  |
| Kiss  et al., 2018^60^ | RCT | Heart | IRI | Adult (12–15 weeks) male  SD rats | 10 | Ar/O_2_/N_2_ 50/21/29  for 3 cycles of 5 min | N_2_/O_2_ 79/21  for 3 cycles of 5 min |  |  | **✔** |  | ✔ |  |  | **+** |  | **+** |

*Abbreviations. Ar, Argon; CO_2_, Carbon dioxide; CCI, Controlled cortical impact ; CHI, Close head traumatic brain injury; CT, controlled trial; CTR, control; EXP, experimental; ICR, Institute of Cancer Research; IRI, ischemia reperfusion injury; KCI, Potassium Chloride; LAD, left anterior descending coronary artery; MCAO, middle cerebral artery occlusion; MODS, multiple organ dysfunction syndrome; MTH, Mild Therapeutic Hypothermia,; N_2_, nitrogen; NA, not available; O_2_, oxygen; pMCAO, permanent middle cerebral artery occlusion ; post-I, post injury; pre-I, pre injury; RCT, randomised controlled trial; SAH, subarachnoid haemorrhage; SD, Sprague-Dawley; tMCAO, Transient middle cerebral artery occlusion; VF, ventricular fibrillation.*

*Symbols. ✔, secondary aims; ✔, primary study aim; +, positive/supporting; =, neutral; -, negative/opposite.*

**Table S9. Risk of bias summary for *in vivo* studies included in the meta-analysis**

| **Study**  **(1^st^ author, year)** | **Selection  Bias** | | | **Performance  Bias** | | **Detection  Bias** | | **Attrition  Bias** | **Reporting  Bias** | **Other Bias** | **Total** |
| --- | --- | --- | --- | --- | --- | --- | --- | --- | --- | --- | --- |
|  | **Sequence generation** | **Baseline Characteristic** | **Allocation concealment** | **Random housing** | **Blinding** | **Random outcome assessment** | **Blinding** | **Incomplete outcome data** | **Selective outcome reporting** | **Other sources of bias** |  |
| Ryang et al., 2011^24^ | + | + | + | ? | ? | ? | + | - | + | + | 6 |
| David et al., 2012^5^ | - | - | + | ? | ? | ? | - | - | + | + | 3 |
| Zhuang et al., 2012^25^ | + | - | - | ? | ? | ? | - | - | + | + | 3 |
| Brucken et al., 2013^26^ | + | + | + | ? | ? | ? | + | + | + | + | 7 |
| Brucken et al., 2014^27^ | + | + | + | ? | ? | ? | + | + | + | + | 7 |
| Fahlenkamp et al., 2014^28^ | + | - | - | ? | ? | ? | + | - | + | + | 4 |
| Ristagno et al., 2014^30^ | - | + | - | ? | ? | ? | + | + | + | + | 5 |
| Brucken et al., 2015^32^ | + | + | + | ? | ? | ? | + | + | + | + | 7 |
| Hollig et al., 2016^35^ | + | + | + | ? | ? | ? | + | - | + | + | 6 |
| Zhao et al., 2016^13^ | + | - | - | ? | ? | ? | + | ? | + | + | 4 |
| Zuercher et al., 2016^36^ | + | + | + | ? | ? | ? | + | + | + | + | 7 |
| Liu et al., 2019^41^ | + | + | + | ? | ? | ? | + | + | + | + | 7 |
| Fumagalli et al., 2020^44^ | + | + | - | ? | ? | ? | + | + | + | + | 6 |
| Kremer et al., 2020^42^ | + | - | - | ? | ? | ? | - | + | + | + | 4 |
| Creed et al., 2021^46^ | + | + | - | ? | ? | ? | + | ? | + | + | 5 |
| Moro et al., 2021^45^ | + | - | + | ? | ? | ? | + | ? | + | + | 5 |
| Antonova et al., 2022^50^ | + | + | - | ? | ? | ? | - | - | + | + | 4 |
| Liu et al., 2022^52^ | + | + | + | ? | ? | ? | + | + | + | + | 7 |
| Xue et al., 2023^51^ | + | - | - | ? | ? | ? | + | ? | + | + | 4 |

*Symbols (colour) legend: + (green), low risk of bias: ? (gray), unclear risk of bias; - (yellow), high risk of bias.*

**Table S10. Risk of bias summary for *in vitro* studies included in the meta-analysis**

| **Study**  **(1^st^ author, year)** | **Selection  Bias** | | | **Performance  Bias** | | **Detection  Bias** | | **Attrition  Bias** | **Selective Reporting  Bias** | **Confounding Bias** | **Other Bias** |
| --- | --- | --- | --- | --- | --- | --- | --- | --- | --- | --- | --- |
|  | **Sequence generation** | **Allocation concealment** | **Appropriate comparison group** | **Housing conditions** | **Blinding** | **Exposure characterization** | **Outcome assessing** | **Incomplete outcome data** | **Selective outcome reporting** | **Sources of bias** | **Adherence to study protocol** |
| Jawad et al., 2009^2^ | NA | -- | ++ | ++ | NA | ++ | ++ | NR | ++ | NR | ++ |
| Loetscher et al., 2009^3^ | NA | -- | ++ | ++ | NA | ++ | ++ | NR | ++ | NR | ++ |
| Harris et al., 2013^7^ | NA | -- | + | ++ | NA | ++ | ++ | NR | ++ | NR | ++ |
| Zhao et al., 2016^13^ | NA | -- | ++ | ++ | NA | ++ | ++ | NR | ++ | NR | ++ |
| Grüßer et al., 2017^17^ | NA | -- | - | ++ | NA | + | ++ | NR | ++ | NR | ++ |
| Koziakova et al., 2019^19^ | NA | -- | ++ | ++ | NA | ++ | ++ | NR | ++ | NR | ++ |

*Symbols (colour) legend: ++ (dark green), definitely low risk of bias; + (green), probably low risk of bias; - (yellow), probably high risk of bias; -- (orange), definitely high risk of bias; NR (grey), not reported risk of bias; NA (no colour), not applicable.*

**Additional findings from meta-analysis**

The tables and figures presented below show the results of meta-analysis that were not included in the main text.

**Table S11. Results of meta-analysis on haemodynamic and metabolic outcome (physiological domain)**

| **Study, year** | **SMD (95% CI) *** | **Heterogeneity I^2^ (%)** |
| --- | --- | --- |
| **Mean Arterial Pressure** |  |  |
| Ryang et al., 2011^24^ | 0·0 (-0·84 - 0·84) |  |
| Brucken et al., 2013^26^ | 0·0 (-1·05 - 1·05) |  |
| Brucken et al., 2014^27^ | 0·0 (-0·92 - 0·92) |  |
| Ristagno et al., 2014^30^ | 0·0 (-1·13 - 1·13) |  |
| Brucken et al., 2015^32^ | 0·0 (-0·98 - 0·98) |  |
| Liu et al., 2019^41^ | 1. (-1·05 - 1·05) |  |
| Fumagalli et al., 2020^44^ | 0·0 (-0·98 - 0·98) |  |
| Liu et al., 2022 | 0·0 (-1·05 - 1·05) |  |
| **Total°** | **0·00 (0·00 - 0·00)** | **0·0**  *Test of heterogeneity, p=1·0000* |
| **Heart Rate** |  |  |
| Ryang et al., 2011^24^ | 0·00 (-0·84 - 0·84) |  |
| Brucken et al., 2013^26^ | 0·00 (-1·05 - 1·05) |  |
| Brucken et al., 2014^27^ | 0·00 (-0·92 - 0·92) |  |
| Ristagno et al., 2014^30^ | 0·00 (-1·13 - 1·13) |  |
| Brucken et al., 2015^32^ | 0·00 (-0·98 - 0·98) |  |
| Liu et al., 2019^41^ | 0·00 (-1·05 - 1·05) |  |
| Fumagalli et al., 2020^44^ | 0·00 (-0·98 - 0·98) |  |
| Liu et al., 2022^52^ | 0·00 (-1·05 - 1·05) |  |
| **Total°** | **0·00 (0·00 - 0·00)** | **0·0**  *Test of heterogeneity, p=1·0000* |
| **Cardiac Output** |  |  |
| Brucken et al., 2013^26^ | 0·00 (-1·05 - 1·05) |  |
| Brucken et al., 2014^27^ | 0·00 (-0·92 - 0·92) |  |
| Ristagno et al., 2014^30^ | 0·00 (-1·13 - 1·13) |  |
| Brucken et al., 2015^32^ | 0·00 (-0·98 - 0·98) |  |
| Fumagalli et al., 2020^44^ | 0·00 (-0·98 - 0·98) |  |
| **Total°** | **0·00 (0·00 - 0·00)** | **0·0**  *Test of heterogeneity, p=1·0000* |
| **Glucose** |  |  |
| Brucken et al., 2013^26^ | 0·81 (-0·30 - 1·91) |  |
| Brucken et al., 2014^27^ | -0·76 (-1·59 - 0·07) |  |
| Brucken et al., 2015^32^ | -0·13 (-0·97 - 0·72) |  |
| **Total°** | **-0·09 (-2·00 - 1·82)** | **59·9**  *Test of heterogeneity, p=0·0828* |
| **Lactate** |  |  |
| Brucken et al., 2013^26^ | 0·31 (-0·74 - 1·37) |  |
| Brucken et al., 2014^27^ | 0·00 (-0·80 - 0·80) |  |
| Brucken et al., 2015^32^ | -0·16 (-1·01 - 0·69) |  |
| **Total°** | **0·01 (-0·53 - 0·56)** | **0·0**  *Test of heterogeneity, p=0·7892* |
| **pH** |  |  |
| Ryang et al., 2011^24^ | 0·00 (-0·84 - 0·84) |  |
| David et al., 2012^5^ | 0·00 (-0·84 - 0·84) |  |
| Ristagno et al., 2014^30^ | 0·00 (-1·13 - 1·13) |  |
| Liu et al., 2019^41^ | 0·00 (-1·05 - 1·05) |  |
| Fumagalli et al., 2020^44^ | 0·00 (-0·98 - 0·98) |  |
| Liu et al., 2022^52^ | 0·00 (-1·05 - 1·05) |  |
| **Total°** | **0·00 (0·00 - 0·00)** | **0·0**  *Test of heterogeneity, p=1·0000* |
| **Base Excess** |  |  |
| Ristagno et al., 2014^30^ | 0·00 (-1·13 - 1·13) |  |
| Fumagalli et al., 2020^44^ | 0·00 (-0·98 - 0·98) |  |
| **Total°** | 0·00 (0·00 - 0·00) | **0·0**  *Test of heterogeneity, p=1·0000* |

*Abbreviations. CI: confidence interval; I^2^, Higgins inconsistency index; SMD, standardised mean difference.*

** A positive value indicates a more favourable outcome for the Argon treatment group, while a negative value indicates a better outcome for the control group. ° The mean effect was estimated using a random effects model.*

**Table S12. Results of meta-analysis on neurodegeneration outcome, related to the histological domain**

| **Study, year** | **SMD (95% CI) *** | **Heterogeneity I^2^ (%)** |
| --- | --- | --- |
| **Neurodegeneration** |  |  |
| Zhuang et al., 2012^25^ – CV right (hippocampus) | -0·68 (-1·98 - 0·61) |  |
| Zhuang et al., 2012^25^ – CV left (hippocampus) | 0·65 (-0·64 - 1·94) |  |
| Fahlenkamp et al., 2014^28^ – NeuN (cortex) | 0·06 (-0·65 - 0·78) |  |
| Fahlenkamp et al., 2014^28^ – NeuN (penumbra) | -0·01 (-0·73 - 0·70) |  |
| Fahlenkamp et al., 2014^28^ – NeuN (ischemic core) | 0·31 (-0·41 - 1·03) |  |
| Ristagno et al., 2014^30^ – HE | 0·65 (-0·64 - 1·94) |  |
| Hollig et al., 2016^35^ – NeuN | 2·74 ( 1·28 - 4·20) |  |
| Hollig et al., 2016^35^ – NeuN (CA1) | -0·34 (-1·33 - 0·65) |  |
| Hollig et al., 2016^35^ – NeuN (CA3/4) | 0·43 (-0·56 - 1·43) |  |
| Zhao et al., 2016^13^ – Tunel (cortex) | 4·83 ( 2·68 - 6·98) |  |
| Zuercher et al., 2016^36^ – FJB (hippocampus) | -0·02 (-0·93 - 0·88) |  |
| Zuercher et al., 2016^36^ – CV (CA1 pyknotic cells) (hippocampus) | 0·51 (-0·41 - 1·42) |  |
| Zuercher et al., 2016^36^ – CV (CA1 cell atrophy) (hippocampus) | -1·71 (-2·80 - -0·63) |  |
| Liu et al., 2019^41^ – NeuN (cortex) | 1·21 ( 0·08 - 2·34) |  |
| Liu et al., 2019^41^ – NeuN (sub cortex) | 1·14 ( 0·02 - 2·26) |  |
| Liu et al., 2019^41^ – NeuN (causo putamen) | -0·40 (-1·42 - 0·63) |  |
| Liu et al., 2019^41^ – NeuN (external capsula) | 0·21 (-0·81 - 1·23) |  |
| Liu et al., 2019^41^ – NeuN (inner boundary) | 1·75 ( 0·51 - 3·00) |  |
| Fumagalli et al., 2020^44^ – HE (cortex) | 0·78 (-0·19 - 1·75) |  |
| Fumagalli et al., 2020^44^ – HE (hippocampus) | 0·41 (-0·54 - 1·36) |  |
| Kremer et al., 2020^42^ – HE (CA1) (hippocampus) | 0·40 (-0·80 - 1·61) |  |
| Kremer et al., 2020^42^ – HE (CA2) (hippocampus) | 0·11 (-1·08 - 1·30) |  |
| Kremer et al., 2020^42^ – HE (CA2/3) (hippocampus) | 0·23 (-0·97 - 1·42) |  |
| Kremer et al., 2020^42^ – HE (CA3) (hippocampus) | 0·77 (-0·48 - 2·02) |  |
| Kremer et al., 2020^42^ – HE (hippocampus) | 0·16 (-1·03 - 1·34) |  |
| Kremer et al., 2020^42^ – HE (cortex lateral) | 0·80 (-0·46 - 2·05) |  |
| Kremer et al., 2020^42^ – HE (cortex medial) | 0·03 (-1·15 - 1·22) |  |
| Creed et al., 2021^46^ (70%) – FJB (cortex) | 2·70 ( 1·30 - 4·10) |  |
| Creed et al., 2021^46^ (70%) – FJB (hippocampus) | 0·40 (-0·57 - 1·36) |  |
| Creed et al., 2021^46^ (79%) – FJB (cortex) | -0·50 (-1·40 - 0·39) |  |
| Creed et al., 2021^46^ (79%) – FJB (hippocampus) | -1·65 (-2·69 - -0·61) |  |
| Antonova et al., 2022^50^ – CV (cortex) | -0·46 (-1·22 - 0·29) |  |
| Liu et al., 2022^52^ – NeuN (%)(cortex) | 0·47 (-0·60 - 1·54) |  |
| Liu et al., 2022^52^ – NeuN (cells/mm^2^) (cortex) | 1·16 (-0·00 - 2·32) |  |
| Liu et al., 2022^52^ – NeuN (sub cortex) | 0·63 (-0·45 - 1·72) |  |
| **Total°** | **0·44 (-0·02 - 0·89)** | **Total 73·6**  **(within 41·7 – between 31·9)**  *Test of heterogeneity, p<0·0001* |
| **Excluding outliers°†** | **0·27 (0·05 - 0·50)** | **Total 11·1**  **(within 0·0 – between 11·1)**  *Test of heterogeneity, p<0·0001* |
| **Excluding studies with high risk of bias°‡** | **0·44 (-0·12 - 1·00)** | **Total 75·7**  **(within 60·8 – between 14·9)**  *Test of heterogeneity, p<0·0001* |

*Abbreviations. CI: confidence interval; I^2^, Higgins inconsistency index; SMD, standardised mean difference.*

** A positive value indicates a more favourable outcome for the Argon treatment group, while a negative value indicates a better outcome for the control group.*

*° The mean effect was estimated using a three-level nested random effects model (Level 1-individual units, Level 2-cluster effects, Level 3-studies).*

***†*** *An extreme effect size (outlier) is characterised by a 95% confidence interval that falls outside the 95% confidence interval of the total estimated mean effect.*

*‡ Studies scoring less than 5 in Table S7 indicate a high risk of bias and are excluded from the model.*

**Table S13. Results of meta-analysis on neurodegeneration outcome (histological domain) in hippocampus and cortex**

| **Study, year** | **SMD (95% CI) *** | **Heterogeneity I^2^ (%)** |
| --- | --- | --- |
| **Hippocampus** |  |  |
| Zhuang et al., 2012^25^ – CV right | -0·68 (-1·96 - 0·59) |  |
| Zhuang et al., 2012^25^ – CV left | 0·65 (-0·62 - 1·92) |  |
| Ristagno et al., 2014^30^ – HE | 0·65 (-0·63 - 1·92) |  |
| Hollig et al., 2016^35^ – NeuN | 2·74 (1·38 - 4·11) |  |
| Hollig et al., 2016^35^ – NeuN (CA1) | -0·34 (-1·33 - 0·65) |  |
| Hollig et al., 2016^35^ – NeuN (CA3/4) | 0·43 (-0·56 - 1·42) |  |
| Zuercher et al., 2016^36^ – FJB | -0·02 (-0·93 - 0·88) |  |
| Zuercher et al., 2016^36^ – CV (CA1 pyknotic cells) | 0·51 (-0·41 - 1·42) |  |
| Zuercher et al., 2016^36^ – CV (CA1 cell atrophy) | -1·71 (-2·77 - -0·66) |  |
| Fumagalli et al., 2020^44^ – HE | 0·41 (-0·54 - 1·36) |  |
| Kremer et al., 2020^42^ – HE (CA1) | 0·40 (-0·80 - 1·60) |  |
| Kremer et al., 2020^42^ – HE (CA2) | 0·11 (-1·08 - 1·30) |  |
| Kremer et al., 2020^42^ – HE (CA2/3) | 0·23 (-0·96 - 1·42) |  |
| Kremer et al., 2020^42^ – HE (CA3) | 0·77 (0·46 - 2·00) |  |
| Kremer et al., 2020^42^ – HE | 0·16 (-1·03 - 1·34) |  |
| Creed et al., 2021^46^ (70%) – FJB | 0·40 (-0·56 - 1·36) |  |
| Creed et al., 2021^46^ (79%) – FJB | -1·65 (-2·66 - -0·64) |  |
| **Total°** | **0·14 (-0·34 - 0·63)** | **Total 65·0**  **(within 65·0 - between 0·0)**  *Test of heterogeneity, p=0·0002* |
| **Excluding outliers°†** | **0·26 (-0·06 - 0·57)** | **Total 0·0**  **(within 0·0 - between 0·0)**  *Test of heterogeneity, p=0·9461* |
| **Excluding studies with high risk of bias°‡** | **0·10 (-0·75 - 0·95)** | **Total 80·7**  **(within 80·7 – between 0·0)**  *Test of heterogeneity, p<0·0001* |
| **Cortex** |  |  |
| Fahlenkamp et al., 2014^28^ – NeuN | 0·06 (-0·65 - 0·78) |  |
| Zhao et al., 2016^13^ – Tunel | 4·83 (2·89 - 6·77) |  |
| Liu et al., 2019^41^ – NeuN | 1·21 (0·11 - 2·32) |  |
| Fumagalli et al., 2020^44^ – HE | 0·78 (-0·19 - 1·75) |  |
| Kremer et al., 2020^42^ – HE lateral | 0·80 (-0·44 - 2·03) |  |
| Kremer et al., 2020^42^ – HE medial | 0·03 (-1·15 - 1·22) |  |
| Creed et al., 2021^46^ (70%) – FJB | 2·70 (1·38 - 4·01) |  |
| Creed et al., 2021^46^ (79%) – FJB | -0·50 (-1·39 - 0·39) |  |
| Antonova et al., 2022^50^ – CV | -0·46 (-1·22 - 0·29) |  |
| Liu et al., 2022^52^ – NeuN (%) | 0·47 (-0·59 - 1·53) |  |
| Liu et al., 2022^52^ – NeuN (cells/mm^2^) | 1·16 (0·02 - 2·29) |  |
| **Total°** | **0·94 (-0·15 - 2·03)** | **Total 87·2**  **(within 0·0 – between 87·2)**  *Test of heterogeneity, p<0·0001* |
| **Excluding outliers°†** | **0·28 (-0·27 - 0·83)** | **Total 45·8**  **(within 0·0 – between 45·8)**  *Test of heterogeneity, p=0·0795* |
| **Excluding studies with high risk of bias°‡** | **0·93 (-0·33 - 2·18)** | **Total 75·9**  **(within 0·0 – between 75·9)**  *Test of heterogeneity, p=0·0208* |

*Abbreviations. CI: confidence interval; I^2^, Higgins inconsistency index; SMD, standardised mean difference.*

** A positive value indicates a more favourable outcome for the Argon treatment group, while a negative value indicates a better outcome for the control group.*

*° The mean effect was estimated using a three-level nested random effects model (Level 1-individual units, Level 2-cluster effects, Level 3-studies).*

***†*** *An extreme effect size (outlier) is characterised by a 95% confidence interval that falls outside the 95% confidence interval of the total estimated mean effect.*

*‡ Studies scoring less than 5 in Table S7 indicate a high risk of bias and are excluded from the model.*

**Table S14. Results of meta-analysis on neuroinflammation outcome, related to the histological domain**

| **Study, year** | **SMD (95% CI) *** | **Heterogeneity I^2^ (%)** |
| --- | --- | --- |
| **Neuroinflammation** |  |  |
| Fahlenkamp et al., 2014^28^ – Microglia ext-Iba1 (cortex) | -0·18 (-0·90 - 0·54) |  |
| Fahlenkamp et al., 2014^28^ – Microglia ext-Iba1 (penumbra) | -0·21 (-0·93 - 0·50) |  |
| Fahlenkamp et al., 2014^28^ – Microglia ext-Iba1 (ischemic core) | -0·35 (-1·07 - 0·37) |  |
| Fahlenkamp et al., 2014^28^ – Astrocytes-GFAP (cortex) | -0·05 (-0·77 - 0·66) |  |
| Fahlenkamp et al., 2014^28^ – Astrocytes-GFAP (penumbra) | 0·05 (-0·66 - 0·77) |  |
| Fahlenkamp et al., 2014^28^ – Astrocytes-GFAP | -0·14 (-0·86 - 0·57) |  |
| Ristagno et al., 2014^30^ – Microglia ext-Iba1 | 0·47 (-0·80 - 1·73) |  |
| Liu et al., 2019^41^ – Microglia ext-Iba1 | 1·05 (-0·05 - 2·16) |  |
| Fumagalli et al., 2020^44^ – Microglia act-Iba1 | 0·93 (-0·07 - 1·92) |  |
| Kremer et al., 2020^42^ – Microglia ext-Iba1 (CA1) (hippocampus) | 0·46 (-0·75 - 1·67) |  |
| Kremer et al., 2020^42^ – Microglia ext-Iba1 (CA2) (hippocampus) | -0·32 (-1·52 - 0·88) |  |
| Kremer et al., 2020^42^ – Microglia ext-Iba1 (CA2/3) (hippocampus) | -0·07 (-1·26 - 1·12) |  |
| Kremer et al., 2020^42^ – Microglia ext-Iba1 (CA3) (hippocampus) | 0·60 (-0·63 - 1·82) |  |
| Kremer et al., 2020^42^ – Microglia ext-Iba1 (hippocampus, dentate, gyrus) | 0·57 (-0·65 - 1·80) |  |
| Kremer et al., 2020^42^ – Microglia ext-Iba1 (cortex post lateral) | 0·98 (-0·31 - 2·27) |  |
| Kremer et al., 2020^42^ – Microglia ext-Iba1 (cortex post medial) | 1·40 ( 0·01 - 2·78) |  |
| Kremer et al., 2020^42^ – Microglia ext-Iba1 (corpus callosum) | 0·45 (-0·76 - 1·66) |  |
| Creed et al., 2021^46^ (70%) – Microglia-F4/80 | -1·76 (-2·92 - -0·59) |  |
| Creed et al., 2021^46^ (79%) – Microglia-F4/80 | 0·83 (-0·09 - 1·76) |  |
| Moro et al., 2021^45^ – Microglia ext-Iba1 (hippocampus) | 0·45 (-0·44 - 1·34) |  |
| Moro et al., 2021^45^ – Microglia ext-Iba1 (cortex ipsilateral) | 0·37 (-0·52 - 1·25) |  |
| Moro et al., 2021^45^ – Microglia ext-Iba1 (corpus callosum) | 0·38 (-0·51 - 1·26) |  |
| Moro et al., 2021^45^ – M2 Phenotype (citoprotective)-YM1 | 1·86 (0·77 - 2·94) |  |
| Liu et al., 2022^52^ – Microglia ext-Iba1 | 1·06 (-0·09 - 2·20) |  |
| Xue et al., 2023^51^ – Microglia ext-Iba1 | 5·22 (0·22 - 10·22) |  |
| **Total°** | **0·51 (-0·08 - 1·10)** | **Total 70·0**  **(within 0·0 – between 70·0)**  *Test of heterogeneity, p=0·0009* |
| **Excluding outliers°†** | **0·54 (0·15 - 0·92)** | **Total 36·6**  **(within 0·0 – between 36·6)**  *Test of heterogeneity, p=0·1042* |
| **Excluding studies with high risk of bias°‡** | **0·69 (-0·17 - 1·54)** | **Total 75·2**  **(within 27·7 – between 47·5)**  *Test of heterogeneity, p=0·0003* |

*Abbreviations. CI: confidence interval; I^2^, Higgins inconsistency index; SMD, standardised mean difference.*

** A positive value indicates a more favourable outcome for the Argon treatment group, while a negative value indicates a better outcome for the control group.*

*° The mean effect was estimated using a three-level nested random effects model (Level 1-individual units, Level 2-cluster effects, Level 3-studies).*

***†*** *An extreme effect size (outlier) is characterised by a 95% confidence interval that falls outside the 95% confidence interval of the total estimated mean effect.*

*‡ Studies scoring less than 5 in Table S7 indicate a high risk of bias and are excluded from the model.*

**Table S15. Results of meta-analysis on inflammatory outcome (histological domain) in hippocampus and cortex**

| **Study, year** | **SMD (95% CI) *** | **Heterogeneity I^2^ (%)** |
| --- | --- | --- |
| **Hippocampus** |  |  |
| Ristagno et al., 2014^30^ – Microglia ext-Iba1 | 0·47 (-0·79 - 1·72) |  |
| Creed et al., 2021^46^ (70%) – Microglia-F4/80 | -1·76 (-2·88 - -0·64) |  |
| Creed et al., 2021^46^ (79%) – Microglia-F4/80 | 0·83 (-0·08 - 1·75) |  |
| Fumagalli et al., 2020^44^ – Microglia act-Iba1 | 0·93 (-0·06 - 1·92) |  |
| Kremer et al., 2020^42^ – Microglia ext-Iba1 (CA1) | 0·46 (-0·74 - 1·66) |  |
| Kremer et al., 2020^42^ – Microglia ext-Iba1 (CA2) | -0·32 (-1·52 - 0·87) |  |
| Kremer et al., 2020^42^ – Microglia ext-Iba1 (CA2/3) | -0·07 (-1·26 - 1·12) |  |
| Kremer et al., 2020^42^ – Microglia ext-Iba1 (CA3) | 0·60 (-0·62 - 1·81) |  |
| Kremer et al., 2020^42^ – Microglia ext-Iba1 (with dentate, gyrus) | 0·57 (-0·64 - 1·79) |  |
| Moro et al., 2021^45^ – Microglia ext-Iba1 | 0·45 (-0·44 - 1·33) |  |
| **Total°** | **0·22 (-0·62 - 1·06)** | **Total 65·5**  **(within 0·0 – between 65·5)**  *Test of heterogeneity, p=0·0370* |
| **Excluding outliers°†** | **0·48 (0·05 - 0·91)** | **Total 0·0**  **(within 0·0 – between 0·0)**  *Test of heterogeneity, p=0·8586* |
| **Excluding studies with high risk of bias°‡** | **0·21 (-1·13 - 1·55)** | **Total 76·7**  **(within 38·4 – between 38·4)**  *Test of heterogeneity, p=0·0031* |
| **Cortex** |  |  |
| Fahlenkamp et al., 2014^28^ – Microglia ext-Iba1 | -0·18 (-0·90 - 0·54) |  |
| Fahlenkamp et al., 2014^28^ – Astrocytes-GFAP | -0·05 (-0·77 - 0·66) |  |
| Liu et al., 2019^41^ – Microglia ext-Iba1 | 1·05 (-0·03 - 2·14) |  |
| Kremer et al., 2020^42^ – Microglia ext-Iba1 post lateral | 0·98 (-0·27 - 2·24) |  |
| Kremer et al., 2020^42^ – Microglia ext-Iba1 post medial | 1·40 (0·07 - 2·72) |  |
| Moro et al., 2021^45^ – Microglia ext-Iba1 ipsilateral | 0·37 (-0·52 - 1·25) |  |
| Moro et al., 2021^45^ – M2 Phenotype (citoprotective)-YM1 | 1·86 (0·81 - 2·91) |  |
| Liu et al., 2022^52^ – Microglia ext-Iba1 | 1·06 (-0·06 - 2·18) |  |
| **Total°** | **0·75 (0·03 - 1·46)** | **Total 36·2**  **(within 36·2 – between 0·0)**  *Test of heterogeneity, p=0·0208* |
| **Excluding outliers°†** | *No outliers detected* | **-** |
| **Excluding studies with high risk of bias°‡** | **1·04 (0·01 - 2·09)** | **Total 0·0**  **(within 13·3 – between 42·2)**  *Test of heterogeneity, p=0·2074* |

*Abbreviations. CI: confidence interval; I^2^, Higgins inconsistency index; SMD, standardised mean difference.*

** A positive value indicates a more favourable outcome for the Argon treatment group, while a negative value indicates a better outcome for the control group.*

*° The mean effect was estimated using a three-level nested random effects model (Level 1-individual units, Level 2-cluster effects, Level 3-studies).*

***†*** *An extreme effect size (outlier) is characterised by a 95% confidence interval that falls outside the 95% confidence interval of the total estimated mean effect.*

*‡ Studies scoring less than 5 in Table S7 indicate a high risk of bias and are excluded from the model.*

**Figure S1. Funnel plots of Ar effect estimates from each study, according to the outcome.**

For each outcome (see ***Table S6),*** the funnel plot shows the relationship between the effect size of studies and their standard errors. The asymmetrical distribution suggests potential publication bias. Tests for funnel plot asymmetry were available for outcomes with at least 10 effects included in the meta-analysis (***panel c, m, n***). Of note, there is no direct function to conduct Egger’s test for nested random effects model. Alternatively, we calculated it by using the standard errors of the effect size estimates as a predictor in the meta-regression.

**Panel a**. Funnel plot of meta-analysis on *in vitro* studies for assessing Ar effect on cell protection. **Panel b**. Funnel plot of meta-analysis on *in vivo* studies for assessing Ar effect on neurological outcome. **Panel c**. Funnel plot of meta-analysis on *in vivo* studies for assessing Ar effect on cognitive and locomotor outcomes (test for asymmetry, p=0·5913). **Panel d**. Funnel plot of meta-analysis on *in vivo* studies for assessing Ar effect on PaO_2_. **Panel e**. Funnel plot of meta-analysis on *in vivo* studies for assessing Ar effect on PaCO_2_. **Panel f**. Funnel plot of meta-analysis on *in vivo* studies for assessing Ar effect on MAP. **Panel g**. Funnel plot of meta-analysis on *in vivo* studies for assessing Ar effect on HR. **Panel h**. Funnel plot of meta-analysis on *in vivo* studies for assessing Ar effect on CO. **Panel i**. Funnel plot of meta-analysis on *in vivo* studies for assessing Ar effect on Glucose. **Panel j**. Funnel plot of meta-analysis on *in vivo* studies for assessing Ar effect on Lactate. **Panel k**. Funnel plot of meta-analysis on *in vivo* studies for assessing Ar effect on pH. **Panel l**. Funnel plot of meta-analysis on *in vivo* studies for assessing Ar effect on BE. **Panel m**. Funnel plot of meta-analysis on *in vivo* studies for assessing Ar effect on neurodegeneration (test for asymmetry, p<0·0001). **Panel n**. Funnel plot of meta-analysis on *in vivo* studies for assessing Ar effect on inflammation (test for asymmetry, p=0·0061).


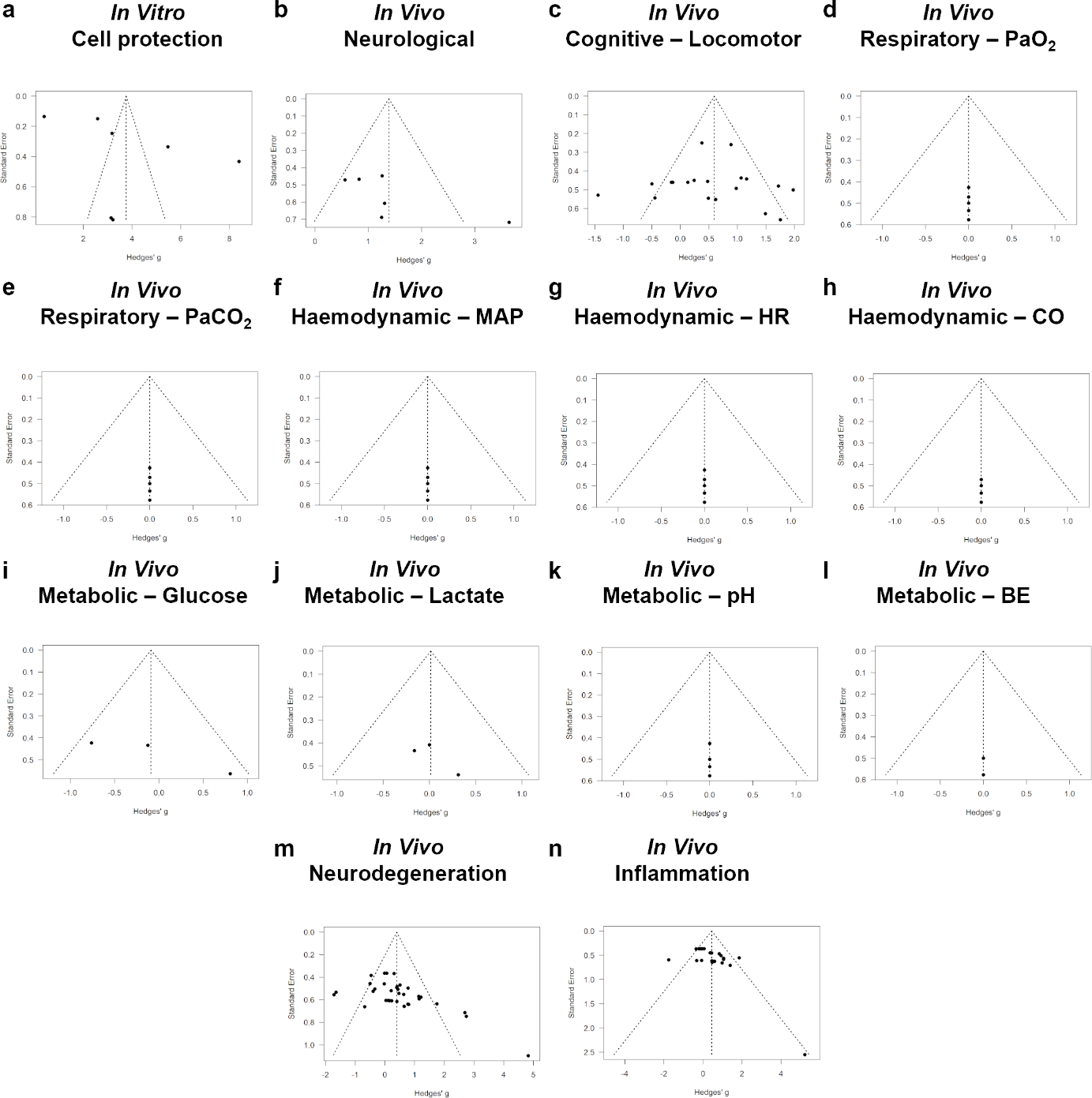


**Figure S2. Forest plot of neurological outcome (functional domain) by percentage of Ar concentration (Ar < 70% and Ar ≥ 70%)**

Data are expressed as SMD and 95% CI. Size of each square represents the study weight in the analysis. The diamond represents the pooled effect from the included studies (the width of the diamond represents the 95% CI for the overall effect) both overall and by subgroups injury models. CIs crossing zero (vertical line) indicate inconclusive results regarding the support for or against Ar.


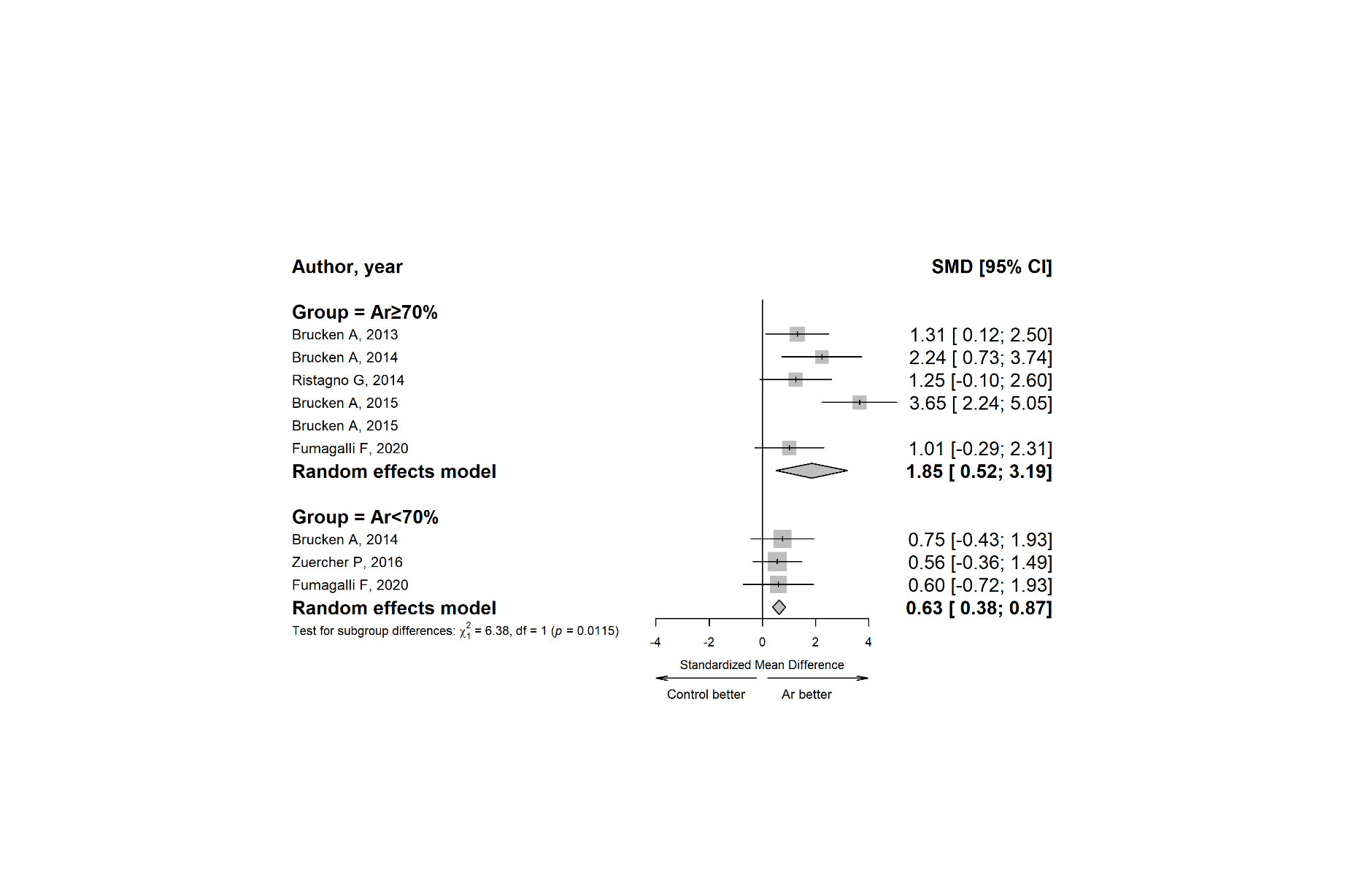


**Figure S3. Forest plot of cognitive and locomotor outcome (functional domain) by percentage of Ar concentration (Ar < 70% and Ar ≥ 70%)**

Data are expressed as SMD and 95% CI. Size of each square represents the study weight in the analysis. The diamond represents the pooled effect from the included studies (the width of the diamond represents the 95% CI for the overall effect) both overall and by subgroups injury models. CIs crossing zero (vertical line) indicate inconclusive results regarding the support for or against Ar.


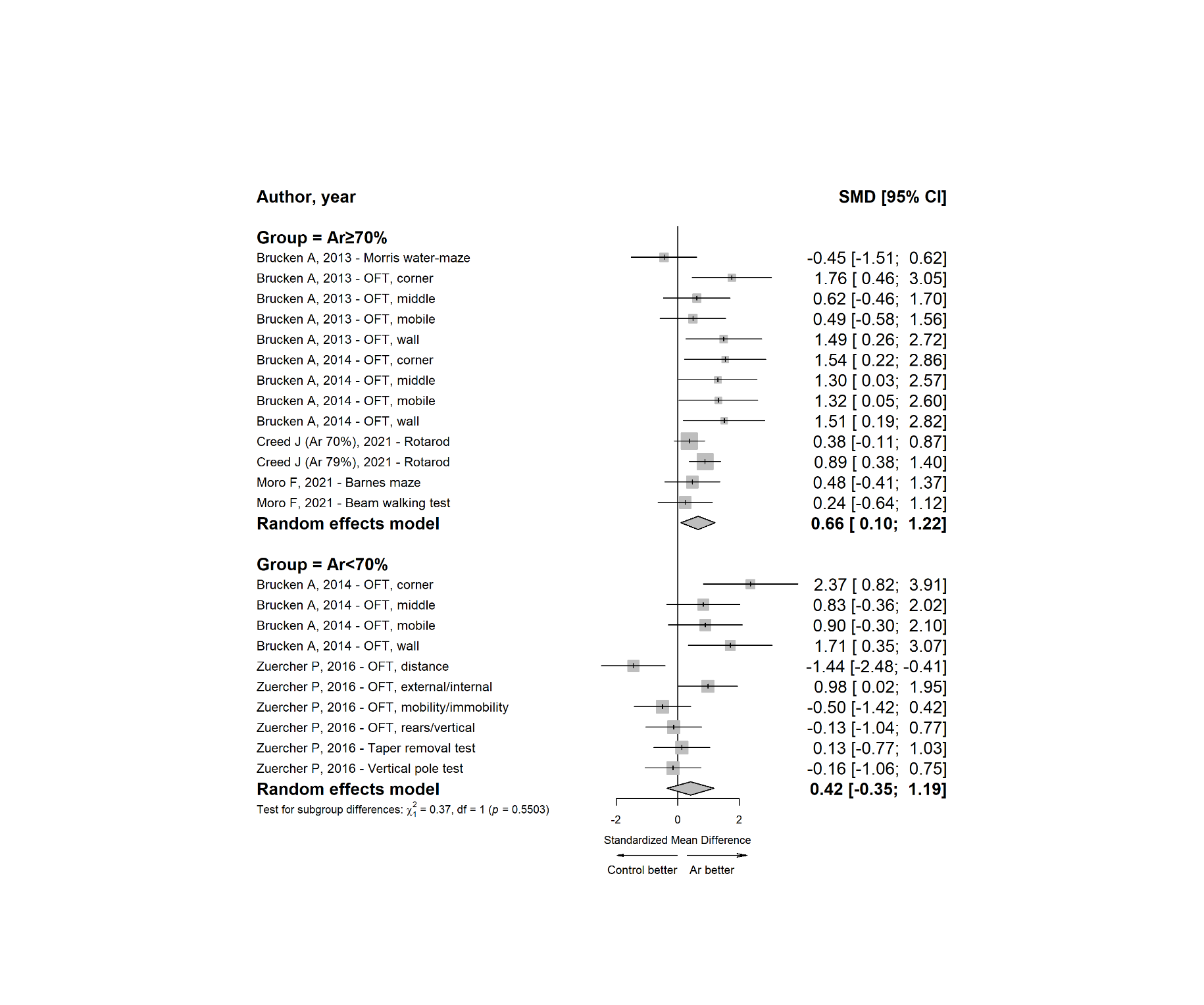


**Figure S4. Forest plot of neurodegeneration outcome (histological domain) by percentage of Ar concentration (Ar < 70% and Ar ≥ 70%)**

Data are expressed as SMD and 95% CI. Size of each square represents the study weight in the analysis. The diamond represents the pooled effect from the included studies (the width of the diamond represents the 95% CI for the overall effect) both overall and by subgroups injury models. CIs crossing zero (vertical line) indicate inconclusive results regarding the support for or against Ar.


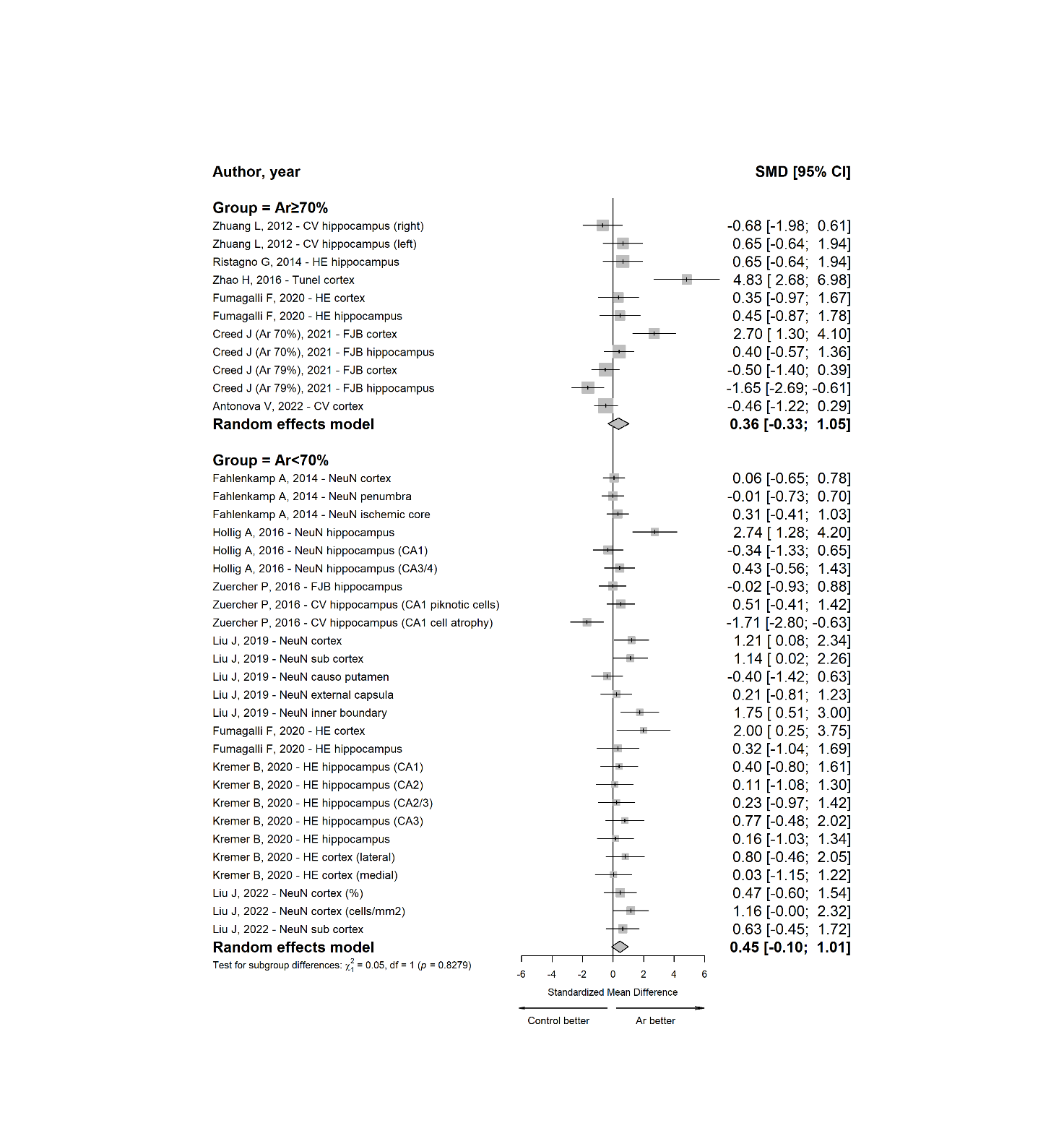


**Figure S5. Forest plot of inflammation outcome (histological domain) by percentage of Ar concentration (Ar < 70% and Ar ≥ 70%)**

Data are expressed as SMD and 95% CI. Size of each square represents the study weight in the analysis. The diamond represents the pooled effect from the included studies (the width of the diamond represents the 95% CI for the overall effect) both overall and by subgroups injury models. CIs crossing zero (vertical line) indicate inconclusive results regarding the support for or against Ar.


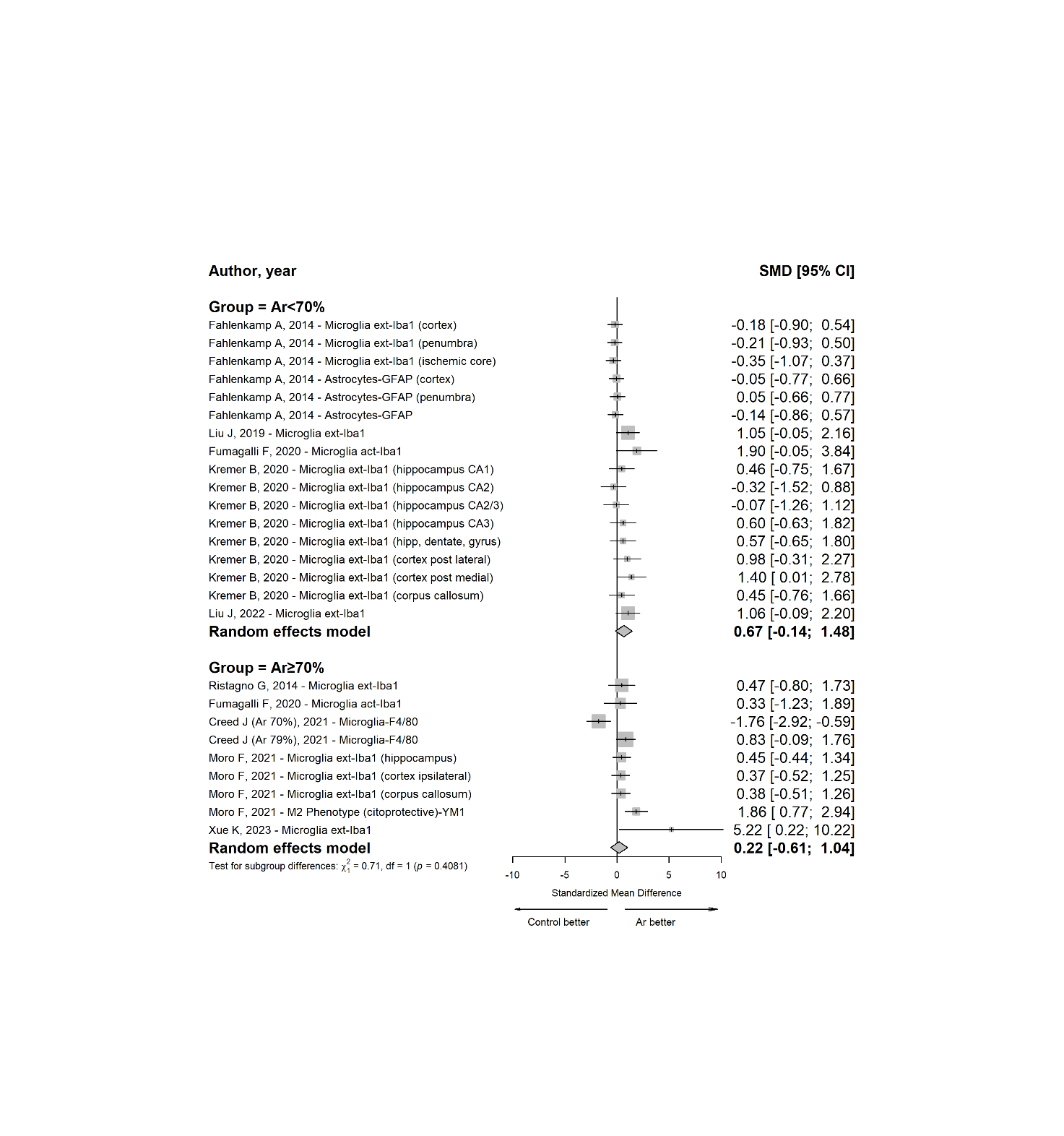


**REFERENCES**

1. Yarin YM, Amarjargal N, Fuchs J, et al. Argon protects hypoxia-, cisplatin- and gentamycin-exposed hair cells in the newborn rat’s organ of Corti. *Hearing Research* 2005; **201**(1-2): 1-9.

2. Jawad N, Rizvi M, Gu J, et al. Neuroprotection (and lack of neuroprotection) afforded by a series of noble gases in an in vitro model of neuronal injury. *Neuroscience Letters* 2009; **460**(3): 232-6.

3. Loetscher PD, Rossaint J, Rossaint R, et al. Argon: neuroprotection in in vitro models of cerebral ischemia and traumatic brain injury. *Critical Care* 2009; **13**(6): R206.

4. Rizvi M, Jawad N, Li Y, Vizcaychipi MP, Maze M, Ma D. Effect of noble gases on oxygen and glucose deprived injury in human tubular kidney cells. *Experimental Biology and Medicine* 2010; **235**(7): 886-91.

5. David HN, Haelewyn B, Degoulet M, Colomb DG, Risso J-J, Abraini JH. Ex vivo and in vivo neuroprotection induced by Argon when given after an excitotoxic or ischemic insult. *PLoS ONE* 2012; **7**(2): e30934.

6. Fahlenkamp AV, Rossaint R, Haase H, et al. The noble gas Argon modifies extracellular signal-regulated kinase 1/2 signaling in neurons and glial cells. *European Journal of Pharmacology* 2012; **674**(2-3): 104-11.

7. Harris K, Armstrong SP, Campos-Pires R, Kiru L, Franks NP, Dickinson R. Neuroprotection against traumatic brain injury by Xenon, but not Argon, is mediated by inhibition at the N-methyl-D-aspartate receptor glycine site. *Anesthesiology* 2013; **119**(5): 1137-48.

8. David HN, Haelewyn B, Risso J-J, Abraini JH. Modulation by the noble gas Argon of the catalytic and thrombolytic efficiency of tissue plasminogen activator. *Naunyn-Schmiedeberg's Archives of Pharmacology* 2013; **386**(1): 91-5.

9. Spaggiari S, Kepp O, Rello-Varona S, et al. Antiapoptotic activity of Argon and Xenon. *Cell Cycle* 2013; **12**(16): 2636-42.

10. Ulbrich F, Kaufmann K, Roesslein M, et al. Argon mediates anti-apoptotic signaling and neuroprotection via inhibition of Toll-like Receptor 2 and 4. *PLOS ONE* 2015; **10**(12): e0143887.

11. Ulbrich F, Lerach T, Biermann J, et al. Argon mediates protection by interleukin‐8 suppression via a TLR2/TLR4/STAT3/NF‐κB pathway in a model of apoptosis in neuroblastoma cells in vitro and following ischemia‐reperfusion injury in rat retina in vivo. *Journal of Neurochemistry* 2016; **138**(6): 859-73.

12. Zhao H, Mitchell S, Koumpa S, et al. Heme oxygenase-1 mediates neuroprotection conferred by Argon in combination with hypothermia in neonatal hypoxia-ischemia brain injury. *Anesthesiology* 2016; **125**(1): 180-92.

13. Zhao H, Mitchell S, Ciechanowicz S, et al. Argon protects against hypoxic-ischemic brain injury in neonatal rats through activation of nuclear factor (erythroid-derived 2)-like 2. *Oncotarget* 2016; **7**(18): 25640-51.

14. Hafner C, Qi H, Soto-Gonzalez L, et al. Argon preconditioning protects airway epithelial cells against hydrogen peroxide-induced oxidative stress. *European Surgical Research* 2016; **57**(3-4): 252-62.

15. Mayer B, Soppert J, Kraemer S, et al. Argon induces protective effects in cardiomyocytes during the second window of preconditioning. *International Journal of Molecular Sciences* 2016; **17**(7): 1159.

16. Lemoine S, Blanchart K, Souplis M, et al. Argon exposure induces postconditioning in myocardial ischemia–reperfusion. *Journal of Cardiovascular Pharmacology and Therapeutics* 2017; **22**(6): 564-73.

17. Grüβer L, Blaumeiser-Debarry R, Krings M, et al. Argon attenuates the emergence of secondary injury after traumatic brain injury within a 2-hour incubation period compared to desflurane: an in vitro study. *Medical Gas Research* 2017; **7**(2): 93.

18. Qi H, Soto-Gonzalez L, Krychtiuk KA, et al. Pretreatment with Argon protects human cardiac myocyte-like progenitor cells from oxygen glucose deprivation-induced cell death by activation of AKT and differential regulation of mapkinases. *Shock* 2018; **49**(5): 556-63.

19. Koziakova M, Harris K, Edge CJ, Franks NP, White IL, Dickinson R. Noble gas neuroprotection: Xenon and Argon protect against hypoxic-ischaemic injury in rat hippocampus in vitro via distinct mechanisms. *British Journal of Anaesthesia* 2019; **123**(5): 601-9.

20. Qi H, Zhang J, Shang Y, Yuan S, Meng C. Argon inhibits reactive oxygen species oxidative stress via the miR-21-mediated PDCD4/PTEN pathway to prevent myocardial ischemia/reperfusion injury. *Bioengineered* 2021; **12**(1): 5529-39.

21. Goebel U, Scheid S, Spassov S, et al. Argon reduces microglial activation and inflammatory cytokine expression in retinal ischemia/reperfusion injury. *Neural Regeneration Research* 2021; **16**(1): 192.

22. Scheid S, Goebel U, Ulbrich F. Neuroprotection is in the air-inhaled gases on their way to the neurons. *Cells* 2023; **12**(20): 2480.

23. Pagel PS, Krolikowski JG, Shim YH, et al. Noble gases without anesthetic properties protect myocardium against infarction by activating prosurvival signaling kinases and inhibiting mitochondrial permeability transition in vivo. *Anesthesia & Analgesia* 2007; **105**(3): 562-9.

24. Ryang Y-M, Fahlenkamp AV, Rossaint R, et al. Neuroprotective effects of Argon in an in vivo model of transient middle cerebral artery occlusion in rats. *Critical Care Medicine* 2011; **39**(6): 1448-53.

25. Zhuang L, Yang T, Zhao H, et al. The protective profile of argon, helium, and xenon in a model of neonatal asphyxia in rats. *Critical Care Medicine* 2012; **40**(6): 1724-30.

26. Brücken A, Cizen A, Fera C, et al. Argon reduces neurohistopathological damage and preserves functional recovery after cardiac arrest in rats. *British Journal of Anaesthesia* 2013; **110**: i106-i12.

27. Brücken A, Kurnaz P, Bleilevens C, et al. Dose dependent neuroprotection of the noble gas argon after cardiac arrest in rats is not mediated by KATP—Channel opening. *Resuscitation* 2014; **85**(6): 826-32.

28. Fahlenkamp AV, Coburn M, De Prada A, et al. Expression analysis following Argon treatment in an in vivo model of transient middle cerebral artery occlusion in rats. *Medical Gas Research* 2014; **4**(1): 11.

29. Ulbrich F, Schallner N, Coburn M, et al. Argon inhalation attenuates retinal apoptosis after ischemia/reperfusion injury in a time- and Ddose-dependent manner in rats. *PLoS ONE* 2014; **9**(12): e115984.

30. Ristagno G, Fumagalli F, Russo I, et al. Postresuscitation treatment with Argon improves early neurological recovery in a porcine model of cardiac arrest. *Shock* 2014; **41**(1): 72-8.

31. Alderliesten T, Favie LMA, Neijzen RW, et al. Neuroprotection by Argon ventilation after perinatal asphyxia: a safety study in newborn piglets. *PLoS ONE* 2014; **9**(12): e113575.

32. Brücken A, Kurnaz P, Bleilevens C, et al. Delayed Argon administration provides robust protection against cardiac arrest-induced neurological damage. *Neurocritical Care* 2015; **22**(1): 112-20.

33. Ulbrich F, Kaufmann KB, Coburn M, et al. Neuroprotective effects of Argon are mediated via an ERK-1/2 dependent regulation of heme‐oxygenase‐1 in retinal ganglion cells. *Journal of Neurochemistry* 2015; **134**(4): 717-27.

34. Broad KD, Fierens I, Fleiss B, et al. Inhaled 45–50% Argon augments hypothermic brain protection in a piglet model of perinatal asphyxia. *Neurobiology of Disease* 2016; **87**: 29-38.

35. Höllig A, Weinandy A, Liu J, Clusmann H, Rossaint R, Coburn M. Beneficial properties of Argon after experimental subarachnoid hemorrhage: early treatment reduces mortality and influences hippocampal protein expression. *Critical Care Medicine* 2016; **44**(7): e520-e9.

36. Zuercher P, Springe D, Grandgirard D, et al. A randomized trial of the effects of the noble gases Helium and Argon on neuroprotection in a rodent cardiac arrest model. *BMC Neurology* 2016; **16**(1): 43.

37. Ulmer TF, Fragoulis A, Dohmeier H, et al. Argon delays initiation of liver regeneration after partial hepatectomy in rats. *European Surgical Research* 2017; **58**(5-6): 204-15.

38. Brücken A, Bleilevens C, Föhr P, et al. Influence of Argon on temperature modulation and neurological outcome in hypothermia treated rats following cardiac arrest. *Resuscitation* 2017; **117**: 32-9.

39. Savary G, Lidouren F, Rambaud J, et al. Argon attenuates multiorgan failure following experimental aortic cross‐clamping. *British Journal of Clinical Pharmacology* 2018; **84**(6): 1170-9.

40. Ma S, Chu D, Li L, et al. Argon inhalation for 24 Hours after onset of permanent focal cerebral ischemia in rats provides neuroprotection and improves neurologic outcome. *Critical Care Medicine* 2019; **47**(8): e693-e9.

41. Liu J, Nolte K, Brook G, et al. Post-stroke treatment with Argon attenuated brain injury, reduced brain inflammation and enhanced M2 microglia/macrophage polarization: a randomized controlled animal study. *Critical Care* 2019; **23**(1): 198.

42. Kremer B, Coburn M, Weinandy A, et al. Argon treatment after experimental subarachnoid hemorrhage: evaluation of microglial activation and neuronal survival as a subanalysis of a randomized controlled animal trial. *Medical Gas Research* 2020; **10**(3): 103.

43. Schmitz SM, Dohmeier H, Stoppe C, et al. Inhaled Argon impedes hepatic regeneration after ischemia/reperfusion injury in rats. *International Journal of Molecular Sciences* 2020; **21**(15): 5457.

44. Fumagalli F, Olivari D, Boccardo A, et al. Ventilation with Argon improves survival with good neurological recovery after prolonged untreated cardiac arrest in pigs. *Journal of the American Heart Association* 2020; **9**(24): e016494.

45. Moro F, Fossi F, Magliocca A, et al. Efficacy of acute administration of inhaled Argon on traumatic brain injury in mice. *British Journal of Anaesthesia* 2021; **126**(1): 256-64.

46. Creed J, Cantillana-Riquelme V, Yan BH, et al. Argon inhalation for 24 h after closed-Head injury does not improve recovery, neuroinflammation, or neurologic outcome in mice. *Neurocritical Care* 2021; **34**(3): 833-43.

47. De Roux Q, Lidouren F, Kudela A, et al. Argon attenuates multiorgan failure in relation with HMGB1 inhibition. *International Journal of Molecular Sciences* 2021; **22**(6): 3257.

48. Schneider FI, Krieg SM, Lindauer U, Stoffel M, Ryang Y-M. Neuroprotective effects of the inert gas Argon on experimental traumatic brain injury in vivo with the controlled cortical impact model in mice. *Biology* 2022; **11**(2): 158.

49. He J, Xue K, Liu J, et al. Timely and appropriate administration of inhaled Argon provides better outcomes for tMCAO mice: a controlled, randomized, and double-blind animal study. *Neurocritical Care* 2022; **37**(1): 91-101.

50. Antonova VV, Silachev DN, Ryzhkov IA, et al. Three-hour Argon inhalation has no neuroprotective effect after open traumatic brain injury in rats. *Brain Sciences* 2022; **12**(7): 920.

51. Xue K, Qi M, She T, et al. Argon mitigates post-stroke neuroinflammation by regulating M1/M2 polarization and inhibiting NF-κB/NLRP3 inflammasome signaling. *Journal of Molecular Cell Biology* 2023; **14**(12): mjac077.

52. Liu J, Veldeman M, Höllig A, et al. Post-stroke treatment with Argon preserved neurons and attenuated microglia/macrophage activation long-termly in a rat model of transient middle cerebral artery occlusion (tMCAO). *Scientific Reports* 2022; **12**(1): 691.

53. Silachev D, Boeva E, Yakupova E, et al. Positive neuroprotective effect of Argon inhalation after photochemically induced ischemic stroke model in rats. *Bulletin of Experimental Biology and Medicine* 2023; **176**(2): 143-9.

54. Irani Y, Pype JL, Martin AR, et al. Noble gas (Argon and Xenon)-saturated cold storage solutions reduce ischemia-reperfusion injury in a rat model of renal transplantation. *Nephron Extra* 2012; **1**(1): 272-82.

55. Faure A, Bruzzese L, Steinberg J-G, et al. Effectiveness of pure argon for renal transplant preservation in a preclinical pig model of heterotopic autotransplantation. *Journal of Translational Medicine* 2016; **14**(1): 40.

56. Martens A, Montoli M, Faggi G, et al. Argon and xenon ventilation during prolonged ex vivo lung perfusion. *Journal of Surgical Research* 2016; **201**(1): 44-52.

57. Martens A, Ordies S, Vanaudenaerde B, et al. A porcine ex vivo lung perfusion model with maximal Argon exposure to attenuate ischemia-reperfusion injury. *Medical Gas Research* 2017; **7**(1): 28.

58. Smith SF, Adams T, Hosgood SA, Nicholson ML. The administration of Argon during ex vivo normothermic perfusion in an experimental model of kidney ischemia-reperfusion injury. *Journal of Surgical Research* 2017; **218**: 202-8.

59. De Deken J, Rex S, Lerut E, et al. Postconditioning effects of Argon or Xenon on early graft function in a porcine model of kidney autotransplantation. *British Journal of Surgery* 2018; **105**(8): 1051-60.

60. Kiss A, Shu H, Hamza O, et al. Argon preconditioning enhances postischaemic cardiac functional recovery following cardioplegic arrest and global cold ischaemia. *European Journal of Cardio-Thoracic Surgery* 2018; **54**(3): 539-46.
